# VEGFR1 promotes cell migration and proliferation through PLCγ and PI3K pathways

**DOI:** 10.1038/s41540-017-0037-9

**Published:** 2017-12-19

**Authors:** Jared C. Weddell, Si Chen, P. I. Imoukhuede

**Affiliations:** Department of Bioengineering, University of Illinois at Urbana-Champaign, Urbana, IL 61801 USA

## Abstract

The ability to control vascular endothelial growth factor (VEGF) signaling offers promising therapeutic potential for vascular diseases and cancer. Despite this promise, VEGF-targeted therapies are not clinically effective for many pathologies, such as breast cancer. VEGFR1 has recently emerged as a predictive biomarker for anti-VEGF efficacy, implying a functional VEGFR1 role beyond its classically defined decoy receptor status. Here we introduce a computational approach that accurately predicts cellular responses elicited via VEGFR1 signaling. Aligned with our model prediction, we show empirically that VEGFR1 promotes macrophage migration through PLC_γ_ and PI3K pathways and promotes macrophage proliferation through a PLC_γ_ pathway. These results provide new insight into the basic function of VEGFR1 signaling while offering a computational platform to quantify signaling of any receptor.

## Thought experiment: discovered the protein functions with mathematics

Many diseases and cancers have the potential to be prevented or cured with treatments that manage the function of specific biological proteins. Developing successful treatments are made difficult due to a lack of understanding of the function of these biological proteins, especially since they can have different functions depending on the type of cell they are functioning in. In this study,  Prof. Imoukhuede and his team from University of Illinois at Urbana-Champaign develop a mathematical approach to understand the overall function of such biological proteins. They apply this approach to a specific biological protein, which is poorly understood, to identify its basic function and discuss why its function may be different in different cells. Overall, this work provides an approach to direct disease treatment development by providing a better understanding of proteins driving the disease.

## Introduction

Vascular endothelial growth factor (VEGF) plays a critical role in many pathologies, including vascular disease and cancer.^1–5^ Despite this role, VEGF-targeted therapies are not clinically effective for many patients.^6,7^ As such, there is an urgent need to develop a better understanding of how VEGF-promoted pathologies can be controlled, mechanistically, to improve the efficiency and specificity of current VEGF treatments.

VEGF receptor-1 (VEGFR1) has emerged as a predictive biomarker for anti-VEGF therapeutics in cancer,^8–10^ but its signaling mechanisms and function remain incompletely defined. VEGFR1 is conventionally described as a decoy receptor that does not produce intracellular signals, due to its high VEGF affinity but low phosphorylation compared to VEGFR2.^11^ However, emerging evidence suggests an active VEGFR1 signaling role: membrane VEGFR1 is upregulated during vascular reperfusion stages in ischemic tissue,^12^ in both hypoxic tumor cells and tumor endothelial cells,^13^ and mice lacking VEGFR1 signaling exhibit reduced tumor vascularization.^14^ Furthermore, VEGFR1 demonstrates tumor activity via placental growth factor (PlGF);^15,16^ PlGF inhibition has shown promise to prevent tumor growth and metastasis.^17^ Given such emerging evidence, we believe that VEGFR1 must have an important signaling role, and we aim to delineate it.

VEGFR1 signaling can be characterized by systems biology to mathematically define receptor signaling mechanisms. The power of this mechanistic approach is its faithfulness to the biological structure of the receptor. Toward this end, the two key signaling mechanisms post-VEGFR1 ligation include: (1) carboxy-terminal VEGFR1 phosphorylation at specific tyrosine sites and (2) adapter binding at these sites. We define these as the key steps because they structurally facilitate the second messenger signaling that directs the angiogenic hallmarks of cell proliferation and migration;^18–20^ as such, these steps may together predict those hallmarks. Indeed, there is evidence that tyrosine site phosphorylation is linked to cell response: cell proliferation results from phosphorylation at the VEGFR2 Tyr^1175^ site, whereas phosphorylation at the VEGFR2 Tyr^1214^ site is linked to cell migration.^18^ Cell responses are similarly associated with adapter binding and phosphorylation at receptor phosphor-tyrosine sites:^21–25^ receptor-induced phosphoinositide-3 kinase (PI3K)-p85α/γ regulatory subunit phosphorylation (hence simply called PI3K phosphorylation) is known to result in cell migration.^25^ While these tyrosine site- and adapter-based approaches are useful to predict cell response, they are often analyzed separately, which does not enable a unified understanding of how RTK structure directs cell function.^26,27^ Therefore, computational models that integrate adapter binding and phosphorylation at specific receptor tyrosine sites would advance structure-based predictions of VEGFR1 signaling.

Here we predict how VEGFR1 directs cell responses by developing, comparing, and validating a structure-based model of carboxy-terminal VEGFR activation and a general VEGFR activation model. We validate our modeling approach experimentally by quantifying adapter phosphorylation and cell migration and proliferation stemming from both VEGFR1 and VEGFR2 signaling and computationally parse out VEGFR1 signaling alone to map the VEGFR1 function. The models quantitatively rank adapter protein contributions to VEGFR1-mediated cell migration and cell proliferation. Model comparison reveals how degrees of model complexity affect predictions of receptor activation and cell response. Computational predictions of cell response to drug treatment are validated via functional assays. Together, our modeling approach provides a new, validated tool for structure-based prediction of cell signaling, applied to grant the exigent mapping of VEGFR1.

## Results

### VEGFR1 primarily induces cell migration

**Fig. 1:**
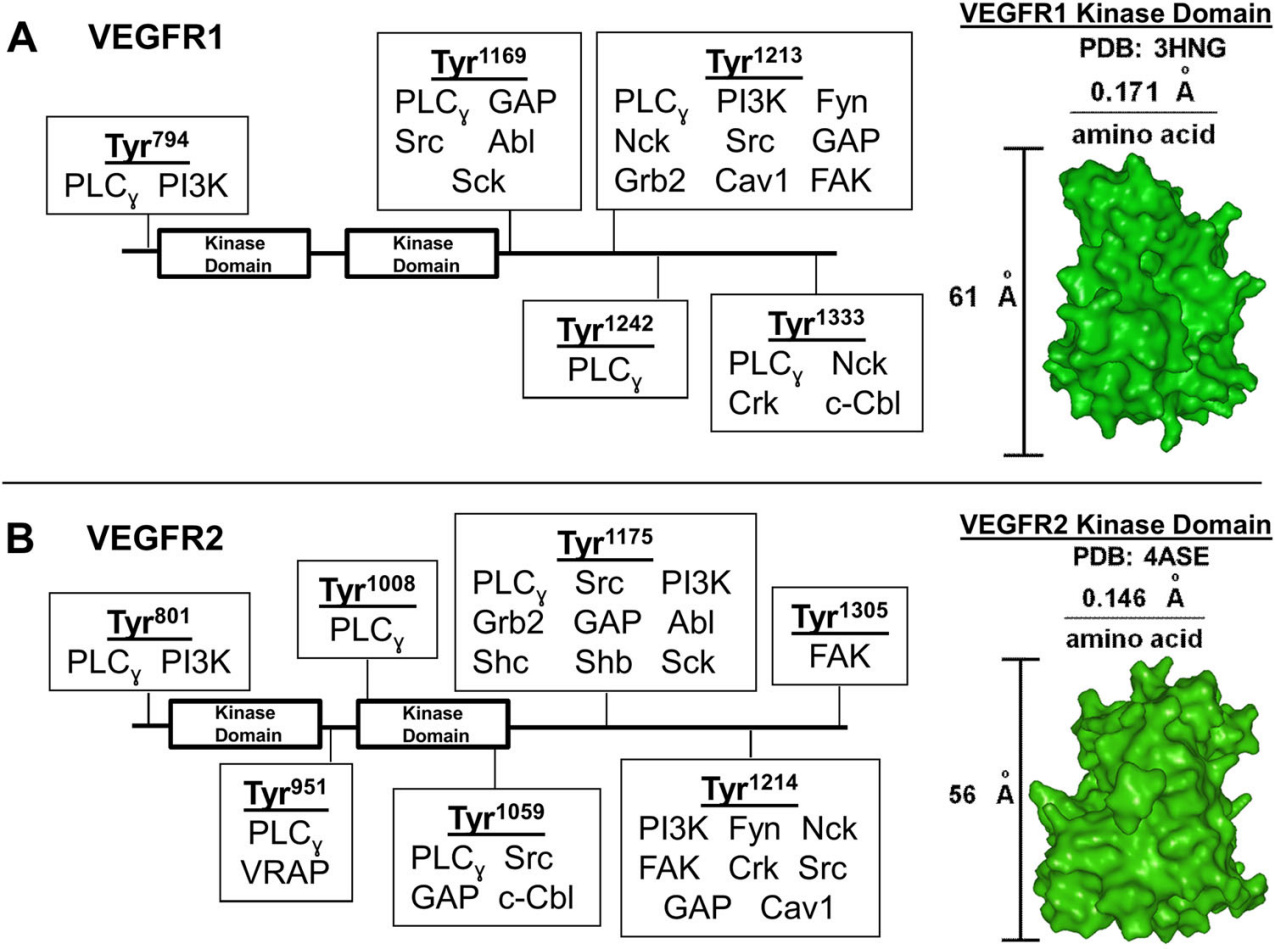
VEGFR–adapter interaction schematics. This schematic depicts the known biology of VEGFR1, VEGFR2, and their related adapters. Adapters bind specific tyrosine (Tyr) sites on **a** VEGFR1 and **b** VEGFR2 (SI Table S4). VEGFR1 and VEGFR2 kinase domain crystal structures were used to measure the distance between individual VEGFR amino acids. This measurement, along with adapter size measurements (SI Table S5), were used to map the adapters and Tyr sites that allow multiple adapters to bind a VEGFR simultaneously, as described in the Supplementary Information

**Fig. 2:**
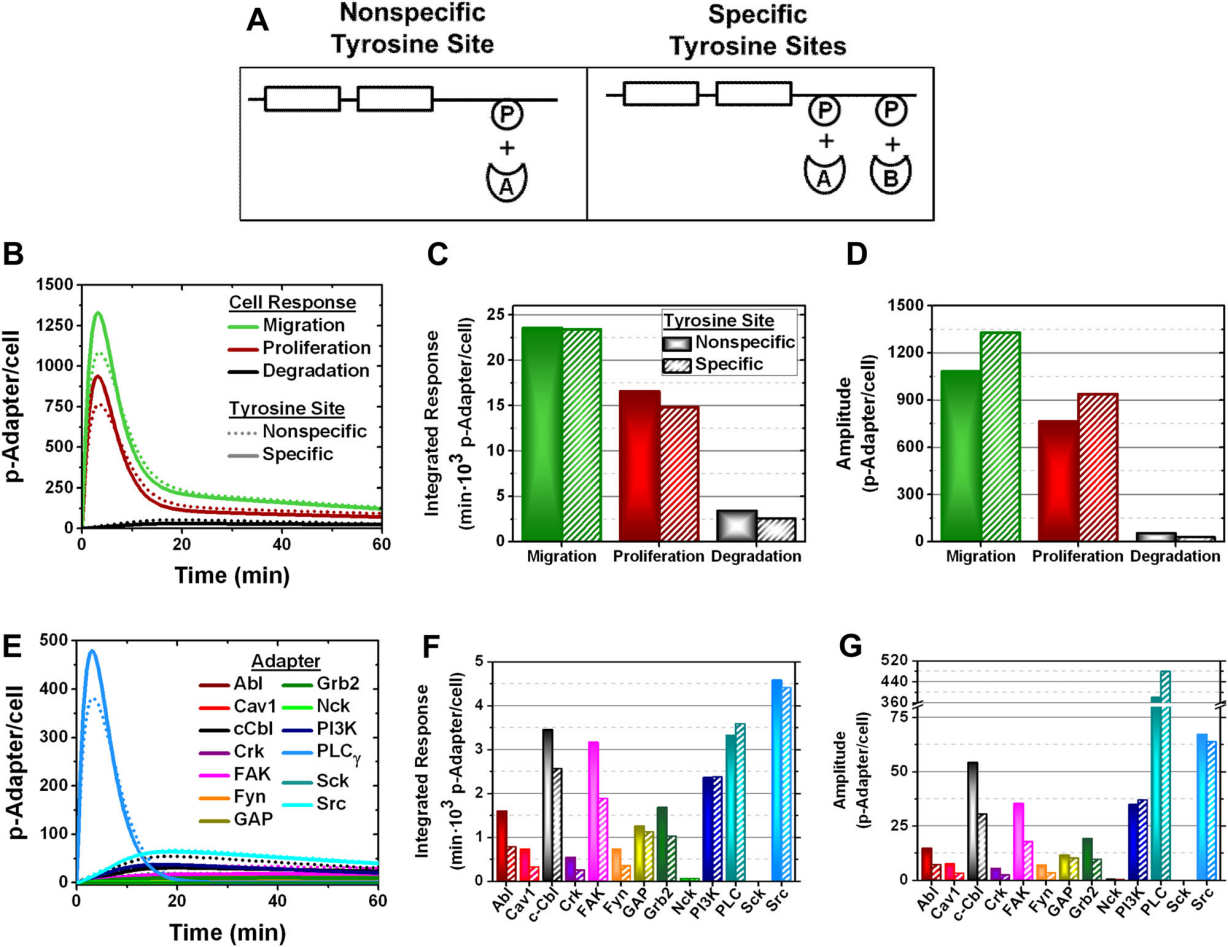
The VEGFR1 structure preferentially activates PLC_γ_ and PI3K. **a** Schematics for the VEGFR–adapter interaction models: (left) adapters bind a single nonspecific VEGFR tyrosine site vs. (right) adapters binding specific VEGFR tyrosine sites. Here adapters are shown in a generalized form, labeled A and B, P represents a phosphorylated receptor Tyr site, and the plus symbol indicates an adapter binding the phosphorylated receptor Tyr site. VEGFR-induced cell responses were modeled by calibrating adapter phosphorylation to each specific cell response, described in the Supplementary Information. HUVEC signaling stemming from VEGFR1 specifically was quantified to determine **b** VEGFR1-induced cell response dynamics, **c** the integrated cell responses (area under the cell response–time curve), and **d** cell response phosphorylation amplitudes. Likewise, **e** VEGFR1-mediated adapter phosphorylation dynamics in HUVECs are analyzed to quantify **f** integrated adapter responses (area under the adapter phosphorylation–time curve) and **g** adapter phosphorylation amplitudes

**Fig. 3:**
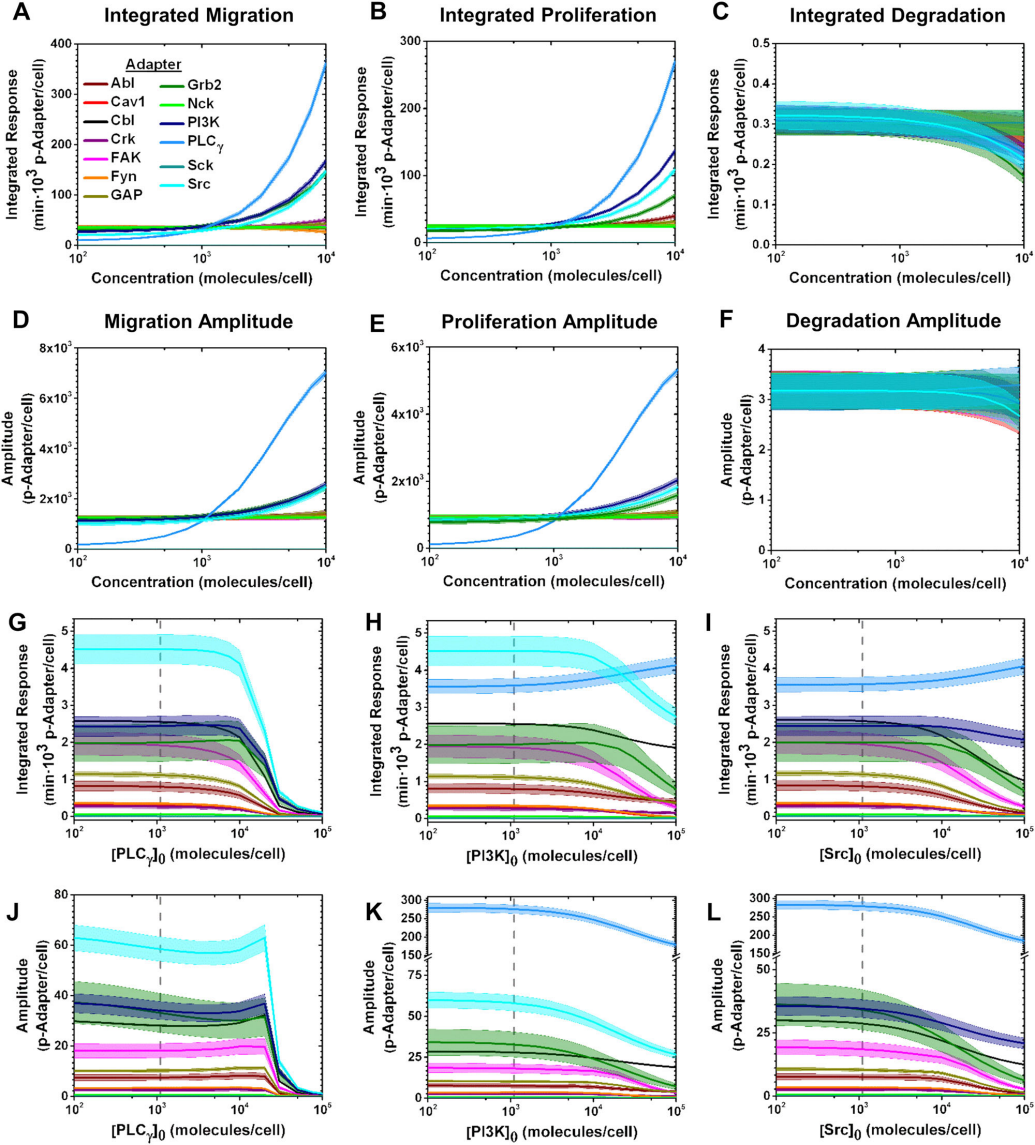
VEGFR1-induced cell responses are primarily directed by PLCγ, PI3K, and Src concentrations. HUVEC **a**–**c** Integrated responses (area under the activation–time curve) and **d**–**f** phosphorylation amplitudes for each cell response, stemming from VEGFR1 signaling only, were quantified with respect to all VEGFR1-associated adapter concentrations, using the specific VEGFR1 tyrosine site model. The **g**–**i** integrated responses and **j**–**l** phosphorylation amplitudes of all adapters were examined with altering **g**,** j** PLCγ concentration, **h**, **k** PI3K concentration, and **i**, **l** Src concentration, using the specific VEGFR1 tyrosine site model. Adapter concentrations were ranged between 10^2^ and 10^5^ molecules/cell. The vertical gray dashed lines indicate the physiological adapter concentration in HUVECs (SI Table S1). Data are presented as mean ± standard deviation given by 4000 Monte Carlo simulations, described in the Supplementary Information

Following VEGF binding, the initial intracellular VEGFR signal transduction steps include: receptor dimerization; autophosphorylation, a post-translational modification (PTM) of carboxy-terminal tyrosines; adapter binding to phospho-tyrosine residues; and adapter phosphorylation. Here we model these receptor signaling mechanisms using mass-action kinetics: specifically, we computationally model VEGF-induced VEGFR phosphorylation, specific adapters binding the VEGFRs, and adapter phosphorylation (Fig. 1). To identify the importance of individual receptor sites in directing aggregated cell responses, we model adapter binding and PTMs occurring non-specifically (nonspecific model) at a single tyrosine site (a common receptor modeling approach) compared to adapter binding and PTMs occurring at specific receptor tyrosine sites (specific model, representing complete receptor physiology) (Fig. 2a). VEGFR-induced cell migration and proliferation were modeled by calibrating adapter phosphorylation to each specific cell response, which interested readers can find a full mathematical description of in the Supplementary Information. To understand the VEGFR1 function, we computationally predict cell signaling stemming from VEGFR1 alone (Figs. 2–3). Both the nonspecific and specific models predict that VEGFR1 primarily induces cell migration (Fig. 2b). This is evidenced by migration exhibiting both the highest integrated cell response (Fig. 2c) and the highest phosphorylation amplitude (Fig. 2d). The specific model reveals mechanistic insight into the migratory cell response: the VEGFR1 tyrosine sites specify cell migration signaling. This is evidenced by the specific model exhibiting a greater contribution to migration signaling; the integrated migration response, relative to proliferation and degradation, increases 16% in the specific model, relative to the nonspecific model (Fig. 2c). Furthermore, the migration phosphorylation amplitude increases 23% in the specific model, relative to the nonspecific model (Fig. 2d). Therefore, we predict that VEGFR1 tyrosine sites are structured to specify cell migration signaling and aim to identify which adapters result in cell migration.

### VEGFR1 tyrosine sites specify PLC_γ_, and PI3K activation through adapter binding competition

VEGFR1 tyrosine sites specify cell migration signaling through phospholipase C-gamma (PLC_γ_) and PI3K phosphorylation (Fig. 2e). PLC_γ_ and PI3K are the only adapters with increased integrated responses (Fig. 2f) and phosphorylation amplitudes (Fig. 2g) between nonspecific and specific models. This unique increase in PLC_γ_ and PI3K activation is due to their binding preference with the VEGFR1 phospho-tyrosine sites (Fig. 1a); only two adapters bind VEGFR1 simultaneously (SI Tables S4 and S5): one adapter at Tyr^794^ and a second adapter at another tyrosine site. PI3K and PLC_γ_ are the only adapters that bind Tyr^794^, thus experiencing less VEGFR1-binding competition than the other adapters, resulting in greater activation. This is evidenced by PLC_γ_ and PI3K activation preferentially occurring at Tyr^794^ (SI Fig S1).

### VEGFR1-promoted cell responses are regulated by coordinated PLC_γ_, PI3K, and Src activation

To predict which adapters primarily direct VEGFR1 cell responses, we perform sensitivity analyses between adapter concentrations and cell responses with the specific site model. We predict that cell proliferation and migration are primarily mediated by PLC_γ_, PI3K, and Src concentrations, in that order (Fig. 3a, b, d, e). Conversely, degradation signaling is not highly altered by adapter concentration (Fig. 3c, f). These three adapters direct VEGFR1 signaling in a coordinated fashion: increasing the PLC_γ_ (Fig. 3g, j), PI3K (Fig. 3h, k), or Src (Fig. 3i, l) concentration to ~2×10^4^ molecules/cell increases phosphorylation of the other two adapters. Increasing PI3K (Fig. 3h) and Src (Fig. 3i) concentrations above ~2×10^4^ molecules/cell increases the PLC_γ_ integrated response, indicating that PI3K and Src promote PLC_γ_ phosphorylation. Together with our result that VEGFR1 is structured to preferentially activate PLC_γ_ and PI3K, we predict that PLC_γ_ and PI3K mediate VEGFR1 cell responses through coordinated activation involving Src.

### Specific tyrosine site modeling captures adapter phosphorylation dynamics

**Fig. 4:**
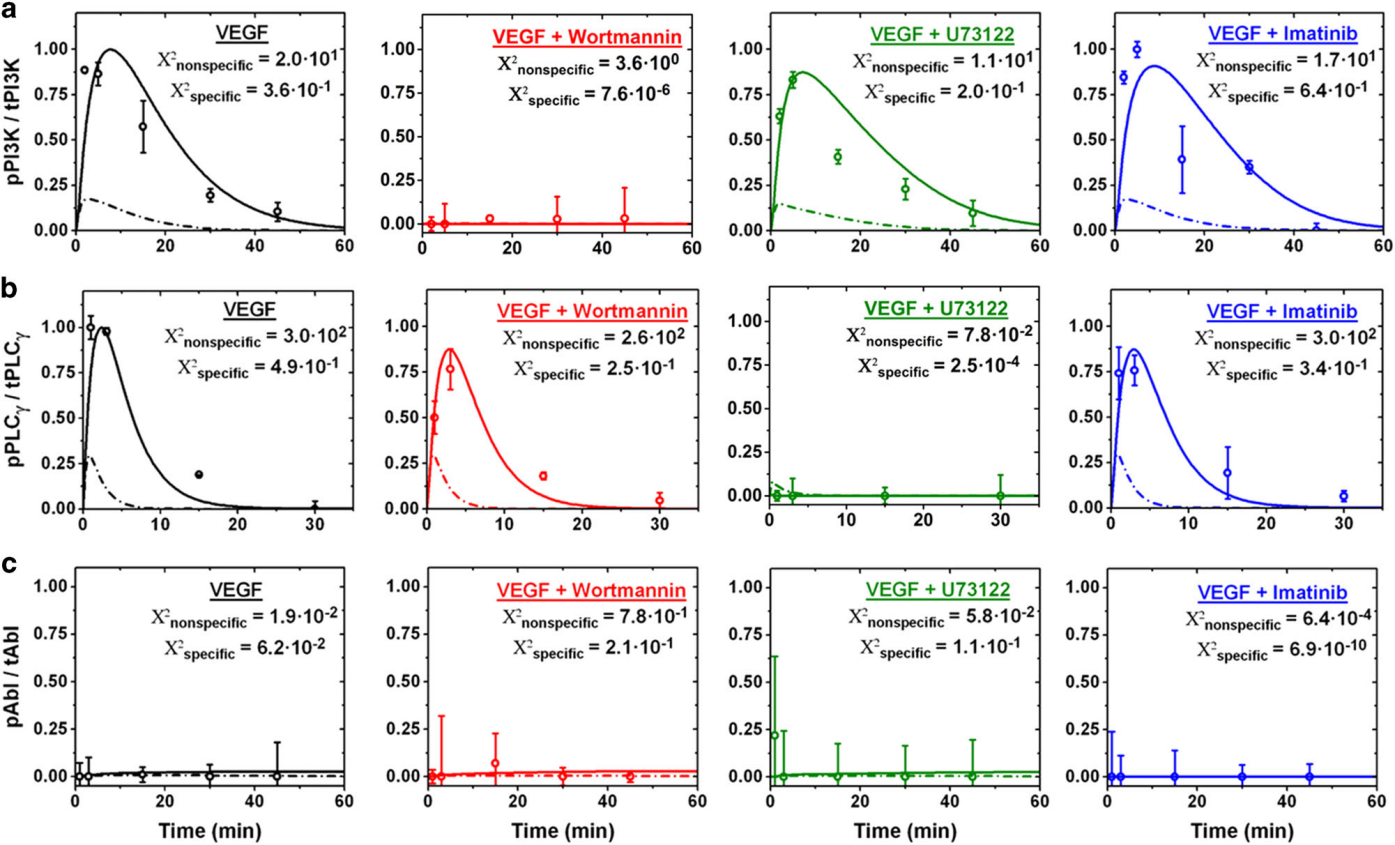
VEGFR1 phosphorylates PI3K and PLCγ with model predicted dynamics. **a** PI3K, **b** PLCγ, and **c** Abl phosphorylation in RAWs were quantified with ELISAs at multiple time points given treatment with VEGF-A164 (50 ng/mL), 100 nM Wortmannin (PI3K inhibitor), 10 µM U73122 (PLCγ inhibitor), and 6 µM Imatinib Mesylate (Abl inhibitor). Data are represented as the mean phosphorylated over mean total protein (p/t) ratio ± standard error of the mean (SEM) for each treatment type and treatment time; here SEM is the sum of the phosphorylated and total protein SEMs. The p/t ratio given inhibitor treatment specific to the protein of interest was subtracted as background for each treatment time. Predicted adapter phosphorylation with modeling a single nonspecific (dashed line) vs. physiologically specific (solid line) VEGFR tyrosine sites are shown compared to experimental data (open circles). Model predictions include adapter phosphorylation contributions from both VEGFR1 and VEGFR2 for validation purposes. Goodness of fit is tested by the Χ^2^ goodness-of-fit test^28^

**Fig. 5:**
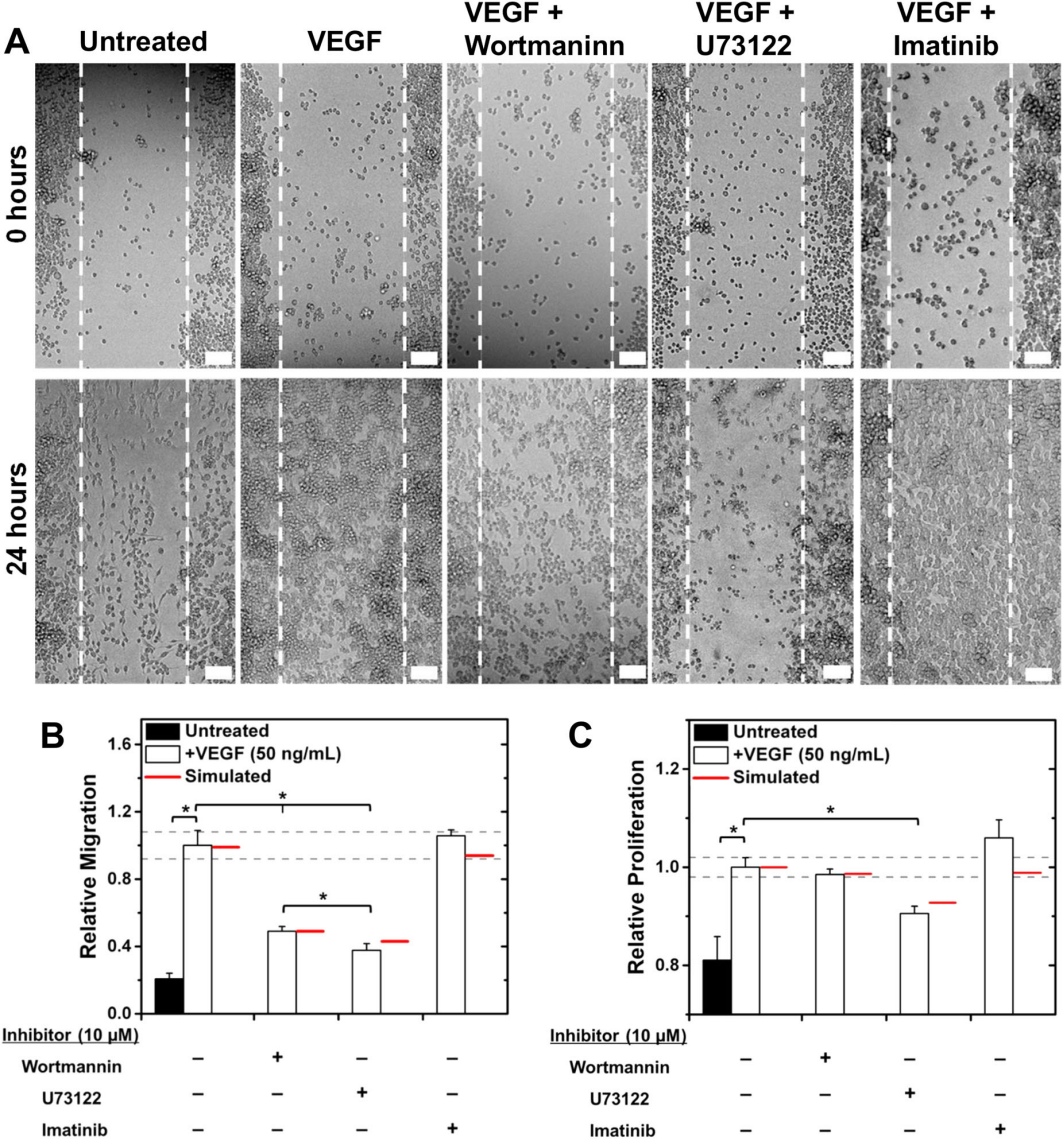
PLCγ and PI3K regulate VEGFR1-induced cell responses in vitro. **a** RAW migration was measured in wound-healing assays at 0 and 24 h post scratch. Scale bars represent 50 µm. **b** Analyzed wound-healing assays show that inhibiting PLCγ or PI3K significantly decreases VEGF-induced RAW migration. **c** PLCγ inhibition significantly decreases VEGF-induced RAW proliferation, measured with MTT assays. Treatments for all experiments were: 50 ng/mL VEGF-A164, 10 µM Wortmannin (PI3K inhibitor), 10 µM U73122 (PLCγ inhibitor), and 10 µM Imatinib Mesylate (Abl inhibitor). All experiments were performed in triplicate, and data are represented as mean ± SEM. Experimental significance is given at *p* < 0.05. (**b**, **c**) The predicted maximum reduction in cell response is given for each inhibitor treatment (red line) using the physiologically specific VEGFR tyrosine ite model. Dashed gray lines outline the range corresponding to 10% variation in cell migration given VEGF treatment alone; inhibitor treatments are predicted to be significant by the model if the predicted cell migration lies outside this range. Model predictions include cell response contributions from both VEGFR1 and VEGFR2 for validation purposes

The specific model accurately predicts PI3K phosphorylation dynamics and magnitude in VEGF-treated RAW 264.7 macrophages, evidenced by the Χ^2^ goodness-of-fit test (Fig. 4a).^28^ All model validations were performed by modeling both VEGFR1 and VEGFR2 signaling (Figs. 4 and 5), as RAWs express both these receptors (SI Fig S3). However, we identify that VEGFR1 signaling dominates VEGF signaling in RAWs computationally (SI Fig S4), and focus on the VEGFR1 signaling contribution. The specific model accurately predicts that PI3K phosphorylation is abrogated by the PI3K-specific inhibitor Wortmannin, while relatively unaffected by inhibiting other adapters (Fig. 4a). Conversely, the nonspecific model accurately predicts relative phosphorylation trends (SI Fig S2) but not phosphorylation magnitudes; the nonspecific model underestimates PI3K phosphorylation by 81% and fails the Χ^2^ goodness-of-fit test (Fig. 4a). Model-predicted PLC_γ_ phosphorylation shows the same trend: the site-specific model accurately predicts PLC_γ_ phosphorylation given VEGF and inhibitor treatments, whereas the nonspecific model fails validation (Fig. 4b). The specific model also accurately identifies which VEGFR1-associated adapters are not critical to VEGFR1 signaling: Abl phosphorylation is not detected as predicted (Fig. 4c). This validation highlights that modeling-specific receptor tyrosine sites is essential to capturing adapter phosphorylation magnitudes and is translatable across cell lines, whereas the conventional approach to model a nonspecific receptor tyrosine site fails physiological validation.

### PI3K and PLC_γ_ are critical to VEGFR1-induced cell migration

We validate our prediction that VEGFR1 promotes cell migration, which is primarily regulated by PLC_γ_ followed by PI3K. We find that VEGFR1 does promote cell migration: VEGF induces significant RAW migration in vitro (Fig. 5a, b). Furthermore, VEGFR1-induced migration is primarily regulated by PLC_γ_, followed by PI3K (Fig. 5a, b). Our specific VEGFR1 tyrosine site model accurately quantifies adapter contributions to RAW migration; RAW migration decreases 79% in vitro with PLC_γ_ inhibition (72% predicted) and 64% with PI3K inhibition (64% predicted) (Fig. 5b). Additionally, our model accurately identifies that Abl is insignificant to VEGFR1-induced migration (Fig. 5b).

### VEGFR1-induced cell proliferation is primarily mediated via PLC_γ_

We validate our prediction that VEGFR1 promotes cell proliferation, primarily through PLC_γ_ activation. VEGFR1 promotes cell proliferation: VEGF induces significant RAW proliferation in vitro (Fig. 5c). We validate our prediction that VEGFR1-induced migration is only significantly regulated by PLC_γ_; RAW proliferation decreases 50% in vitro with PLC_γ_ inhibition (Fig. 5c). Conversely, PI3K and Abl inhibition do not significantly affect cell proliferation, accurately predicted by the specific VEGFR1 site model.

## Discussion

The VEGFR1 status as a decoy receptor may not fully capture its signaling role;^8^ however, few studies have probed VEGFR1 signaling,^11^ which is due to the low phosphorylation levels VEGFR1 exhibits. As tyrosine kinase receptor family is known to signal through coupling with the SH2 domain of adapters,^29^ examining VEGFR1-adapter binding can offer new insight into VEGFR1 signal propagation. To this end, we developed and validated a receptor-adapter interaction modeling approach, which accurately predicts cell responses from adapter phosphorylation, and is translatable across receptor and cell types. Combining this modeling approach with experimental validation identified that VEGFR1 induces macrophage migration via PLC_γ_ and PI3K pathways and induces proliferation via a PLC_γ_ pathway.

### Modeling techniques allow prediction of receptor signaling roles

Our modeling approach quantifies adapter phosphorylation and cell responses simultaneously to map unknown receptor signaling pathways. Our modeling approach integrates the pioneered approaches that accurately predict select adapter–receptor interactions^30–33^ and cell responses^34–36^ from external stimuli. We additionally advance receptor signaling models by providing the ability to map unknown receptor pathways. Furthermore, we show that our approach to model specific receptor tyrosine sites offers physiological relevancy; both nonspecific and specific VEGFR1 tyrosine site models are validated when only the shape of adapter phosphorylation over time is considered (SI Fig S2), but only the specific tyrosine site model accurately predicts adapter phosphorylation magnitudes (Fig. 4). Additionally, our receptor–adapter modeling approach can be easily integrated into pharmacokinetic/pharmacodynamic models that quantify extracellular VEGF dynamics in response to anti-VEGF drugs.^37–39^ One of the major challenges for developing personalized, clinically relevant computational platforms is the difficulty of capturing all relevant physiological processes at multiple scales.^40,41^ By integrating extracellular VEGF dynamics, VEGF–VEGFR interactions, and subsequent intracellular VEGFR signaling, we can provide a clinically relevant platform to explore how anti-VEGF drugs mediate VEGFR signaling simultaneously at the tissue macroscale and intracellular microscale.

### Mapping the native VEGFR1 function requires VEGFR2 signaling

Our modeling approach accurately quantified adapter phosphorylation and cell proliferation and migration in macrophages, which was then extended to map the VEGFR1 function. While macrophages express high VEGFR1, they also lowly express VEGFR2 (SI Fig S3). While our model captures signaling stemming from both VEGFR1 and VEGFR2, the question may arise as to why these experiments were not conducted with VEGFR2 inhibition to measure VEGFR1 signaling alone. The reason is that we seek to map the native function of VEGF-stimulated VEGFR1, which cannot be identified if VEGFR2 signaling is abolished. Indeed, it is well established that VEGFR1 and VEGFR2 expression are inversely related;^12,42–44^ abolishing VEGFR2 signaling would increase VEGFR1 expression, which may affect VEGFR1-adapter binding kinetics, altering adapter phosphorylation and cell response dynamics. Furthermore, VEGFR2 knockdown has been shown to alter basal cell physiology and function, altering cell proliferation potential of unstimulated cells.^42,45^ VEGFR2 knockdown also alters basal levels of total intracellular kinase expression and baseline phosphorylation: VEGFR2 knockdown increases baseline c-Jun phosphorylation in unstimulated cells,^42^ a kinase downstream PI3K that regulates cell proliferation and migration.^46,47^ By contrast, VEGFR2 knockdown decreases baseline phosphorylation of extracellular signal–regulated kinase and Akt,^45^ kinases of downstream adapters such as Src and PI3K that promote cell migration/proliferation.^48^ We expect such adapter and downstream kinase expression alterations from VEGFR2 knockdown to alter VEGFR1 signal propagation and cell response dynamics. Rather than studying VEGFR2 inhibition for these reasons, we computationally parse out the RAW signaling contribution from VEGFR1 compared to VEGFR2, identifying that VEGFR1 signaling dominates VEGF signaling in RAWS; VEGFR1 exhibits a 2.4-fold higher RAW migration, and 2.6-fold higher RAW proliferation, integrated response than VEGFR2. Thus VEGFR2 knockdown presents dynamic changes in cell physiology that may alter the VEGFR1 function, which should be explored in a future study.

### Translating receptor signaling across cell lines advances translational research

Our modeling approach accurately quantified VEGFR1-induced macrophage migration and proliferation through adapter phosphorylation. Our modeling approach required a calibration step where adapters VEGFR1 signals through were identified in endothelial cells (SI Table S3), due to the rich VEGF–VEGFR data available in endothelial cells,^49,50^ and then translated to simulate VEGFR1 signaling in macrophages, which are data poorer in regards to VEGF signaling.^51^ The rationale for this approach is our hypothesis that VEGFR1 is the exact same protein in endothelial cells and macrophages, and thus differential roles VEGFR1 has in these cells must be dependent on the intracellular (adapter expression) and extracellular (VEGFR2 surface expression) environments, as opposed to an inherent difference in VEGFR1 function. Indeed, we show that there is a sizeable difference between VEGFR1-interacting adapter expression (SI Tables S1 and S6) and VEGFR2 expression (SI Table S1, Fig S4) for human umblical vein endothelial cells (HUVECs) and RAW macrophages, agreeing with this hypothesis. We performed this translation across cell lines for two reasons: (1) it allows higher confidence in our model validations, since we are seeding and validating our model from two independent data sources, and (2) it highlights the translatable feature of this modeling approach. Indeed, the ability to translate across cell types has the potential to reduce experimental costs and increase model development time, as the richest data source available can be used even when modeling a new condition. This increased model development time and reduced experimental costs advances translational research by allowing model-informed decisions to be made quicker and with higher confidence.

### qFlow cytometry accurately quantifies membrane receptors

Our ability to accurately quantify VEGFR1 signaling highlights the power of integrating experiment and computation to provide new biology insight: empirical evidence defined VEGFR1–adapter reactions, kinetics, and concentrations for our model, which in turn provided testable VEGFR1 signaling predictions that we confirmed experimentally. This first step, model parameterization, is essential to develop physiologically relevant models, as previously described.^52–54^ We achieved VEGFR concentration parameterization with quantitative flow (qFlow) cytometry,^12,55,56^ a recently established high-throughput approach that detects receptor expression with a fluorescent affinity probe and quantifies absolute receptor concentrations using fluorescent calibration standards.^55^ While qFlow cytometry is becoming an essential tool for parameterizing receptor concentrations in computational models,^8,32,37,39,57–60^ analogous methods for quantifying other receptor signaling parameters, such as adapter phosphorylation rates, are not well established. As such, most computational models contain parameters that are estimated or generalized across multiple species or interactions;^61^ Bose and Janes recently developed one such method for high-throughput characterization of signal molecule dephosphorylation kinetics via phosphatase activity.^62^ Development of such high-throughput methods to completely parameterize receptor signaling models, from species concentrations to specific kinetics for every interaction, would unlock additional options for tuning receptor signaling, such as by targeting specific phosphatases, while maintaining high physiological relevancy.

### VEGFR1 preferentially activates PLCγ in burst activation to induce cell migration and proliferation, possibly through Ca^2+^ signaling

We show that VEGFR1-induced PLC_γ_ activation is required for macrophage migration and proliferation and hypothesize that this VEGFR1-PLC_γ_-mediated migration involves Ca^2+^ signaling. PLC_γ_ phosphorylation is known to activate Ca^2+^ influx^63,64^ in oscillatory bursts.^65–68^ Furthermore, directed cell migration requires Ca^2+^ pulses near the leading edge of the cell.^69–71^ From this prior knowledge, combined with the delta function-like PLC_γ_ activation we observe here, we hypothesize that VEGFR1 phosphorylates PLC_γ_ in quick bursts to induce Ca^2+^ pulses and direct cell migration. This burst PLC_γ_ activation could explain how cells migrate toward a VEGF gradient, with a possible mechanism being as follows: (1) VEGF binds plasma membrane VEGFR1 on the cell facing the gradient; (2) VEGFR1 recruits and phosphorylates PLC_γ_; and (3) phosphorylated PLC_γ_ causes Ca^2+^ pulses by activating Ca^2+^ channels, a well-established mechanism^72–74^ reviewed by Mikoshiba,^75^ initiating migration toward the VEGF gradient. This mechanism is further supported by experimental data showing that Ca^2+^ pulse following VEGF simulation is required for HUVEC migration.^73^ As the extent of directed cell migration is dependent on growth factor gradient patterns,^76^ we hypothesize that VEGFR1-PLC_γ_ activation acts as a VEGF gradient sensor to determine both cell migration direction and magnitude. Additionally, PLC_γ_-Ca^2+^ signaling promotes cell proliferation through downstream activation of protein kinase C^44^. These studies showcase that cell migration and proliferation mechanisms through PLC_γ_-Ca^2+^ signaling have been established. Future work experimentally probing PLCγ-Ca^2+^ signaling through VEGFR1 to mediate cell migration and proliferation is necessary to validate our VEGFR1-PLC_γ_-Ca^2+^ signaling hypothesis.

### Ca^2+^ signaling may indirectly regulate PI3K activation by VEGFR1

We identified PI3K as a primary adapter directing VEGFR1-mediated macrophage migration. Primarily, PI3K is known to promote cell migration through Akt activation,^77,78^ which also involves Ca^2+^ signaling; PI3K/Akt activation translocates Ca^2+^ channels to the cell membrane, inducing Ca^2+^ entry into cells, and subsequent cell migration.^79^ However, PI3K activation does not induce Ca^2+^ signaling in HUVECs;^80^ rather, PI3K is activated by Ca^2+^ to promote HUVEC migration.^81^ Since VEGF–VEGFR–adapter phosphorylation data from HUVECs was used to calibrate our computational model, and validated in macrophages, this indicates that VEGFR1-PI3K may play an important role in indirectly activating Ca^2+^ signaling in macrophages.

### The PLCγ-, PI3K-, and Src-dependent relationship may form a Ca^2+^ signaling regulatory loop

**Fig. 6:**
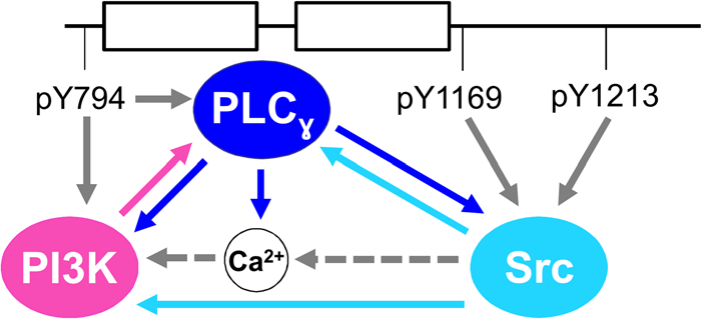
VEGFR1 preferentially activates PLC_γ_, PI3K, and Src, possibly to form a Ca^2+^ signaling regulatory loop. Our simulations predict that VEGFR1 tyrosine sites are structured to preferentially associate with PLC_γ_ or PI3K at Tyr^794^ and Src at Tyr^1169^ or Tyr^1213^, simultaneously, as portrayed. We theorize that this PLC_γ_, PI3K, and Src activation scheme by VEGFR1 forms a Ca^2+^ signaling regulatory loop, as depicted. Arrow color indicates adapter or Ca^2+^ signal activation by VEGFR1 (solid gray), PLC_γ_ (blue), PI3K (pink), Src (cyan), or through Ca^2+^ signaling (dashed gray). Additional VEGFR1-binding sites and adapter association are not shown

We observed a dependent relationship between VEGFR1-induced PLC_γ_, PI3K, and Src phosphorylation. As PI3K and PLC_γ_ cooperate to initiate Ca^2+^ signaling,^82^ we hypothesize that PI3K, PLC_γ_, and Ca^2+^ have a dependent relationship to robustly mediate VEGFR1-induced cell migration and proliferation. Furthermore, PLC_γ_-induced Ca^2+^ signaling phosphorylates Src,^83^ and Src phosphorylates PLC_γ_^80,83,84^ and PI3K.^85–87^ Thus we hypothesize from these studies and our results that VEGFR1 is structured to preferentially activate a PLC_γ_, PI3K, and Src regulatory loop mediating Ca^2+^ signaling (Fig. 6) and subsequent cell migration and proliferation.

### VEGFR1-promoted hematopoietic progenitor cell migration may be required for tumor cell metastasis

The strong VEGFR1 migratory signal we identify here indicates that VEGFR1 signaling may be required for hematopoietic progenitor cell (HPC) migration to form premetastatic niche clusters. Metastasis from the primary tumor site requires circulating tumor cells to extravaste into secondary sites.^88^ Prior to this process, the tumor primes premetastatic niches, sites receptive to recruiting circulating tumor cells, to direct at which secondary sites metastasis occurs.^89^ These premetastatic niches are characterized by clustering of VEGFR1-positive HPCs; inhibiting VEGFR1 on HPCs prevents premetastatic niche formation and tumor cell metastasis.^90^ This effect of premetastatic niche formation being prevented with VEGFR1 inhibition may be explained by HPC migration requiring VEGFR1 signaling; thus inhibiting VEGFR1 signaling would prevent HPC migration, HPC clustering, and subsequent tumor cell metastasis. Furthermore, Akt activation has been implicated in macrophage-assisted cancer cell invasion,^91^ supporting our hypothesis that VEGFR1-PI3K-Ca^2+^ signaling (Fig. 6) promotes macrophage migration. Therefore, targeting VEGFR1-induced HPC migration may be a therapeutic option to prevent tumor cell metastasis.

### Receptor signaling can be comprehensively quantified by modeling adapter–adapter interactions and specific phosphatases

Our modeling approach accurately and quantitatively predicted adapter phosphorylation and cell responses through complex formation between specific VEGFR1 tyrosine sites and single adapters, with adapter dephosphorylation occurring through a generalized phosphatase. Building upon this validated model to include adapter–adapter interactions and specific phosphatases would comprehensively represent VEGFR1 signaling. Modeling adapter–adapter interactions would identify how VEGFR1 signaling is directed through adapter cooperativity; adapter–adapter interactions occur via adapter SH3 domains^92^ to form larger signaling complexes that direct differential cell outcomes.^93,94^ Our ability to accurately model multi-adapter complex formation with VEGFR1 is currently limited, as no known experimental or computational studies have mapped the adapter–adapter interactions downstream of VEGFR1. This limitation may be overcome by identifying VEGFR1-associated adapter–adapter interactions from VEGF-induced protein phosphorylation dynamics, a predictive approach validated with the epidermal growth factor receptor signaling axis.^95^

Modeling specific phosphatases would identify additional VEGFR1-targeting therapeutics; since different phosphatases bind specific adapters to dynamically regulate receptor signaling,^96^ VEGFR1-induced adapter phosphorylation and cell responses could be directed by targeting specific phosphatases. Our ability to model specific phosphatases is currently limited however, as the specific phosphatases involved in VEGFR1 signaling, and their adapter interaction kinetics, have not been determined. This limitation may be overcome using the high-throughput assay for identifying phosphoprotein-specific phosphatases and kinetics developed by the Janes' laboratory.^62^ Overall, incorporating adapter–adapter interactions and phosphatase specificity into our VEGFR1 model would provide further insight into how VEGFR1 signaling is directed systemically and identify additional proteins or interactions that can be targeted to tune VEGFR1 signaling.

### Conclusions

Our modeling approach has identified that VEGFR1 actively promotes macrophage migration and proliferation primarily via the PLC_γ_ and PI3K pathways and has posited a new hypothesis that adapter coordination and Ca^2+^ signaling may regulate this VEGFR1-mediated migratory response. These findings critically advance our understanding of VEGF signaling by providing a structurally based mechanism for VEGFR1 function. Our findings and our modeling platform also offer mechanistic guidance for developing therapeutics targeting VEGFR1 signaling. This also represents a paradigm shift, since VEGF, generally, and VEGFR2 are primary targets for drug discovery. This modeling approach provides a foundation to fully understand signaling mechanisms for any receptor, an essential step to develop effective therapeutics for a wealth of pathologies.

## Methods

Here we provide a brief overview of the computational and experimental methods. Interested readers can find complete description of the computational model, including parameters, and experimental procedures, including vendor information, in the Supplementary Information.

### Computational models

VEGFR–adapter interaction models are defined by mass-action kinetics using ordinary differential equations and solved with the SimBiology toolbox in MATLAB. In general, the VEGFR–adapter scheme interaction scheme follows:

$$\begin{array}{c}{\rm{VEGF + VEGFR}}\ \mathop{\rightleftarrows}\limits_{{{\rm{koff}}_{{\rm{VEGF}}}}}^{{{\rm{kon}}_{{\rm{VEGF}}}}}\ {\rm{pVEGFR}}\\ {\rm{pVEGFR + A}}\ \mathop{\rightleftarrows}\limits_{{{\rm{koff}}_{\rm{A}}}}^{{{\rm{kon}}_{\rm{A}}}}\ \left[ {{\rm{pVEGFR:A}}} \right]\\ \left[ {{\rm{pVEGFR:A}}} \right]\mathop{\longrightarrow}\limits^{{{\rm{kp}}}}\left[ {{\rm{pVEGFR:pA}}} \right]\\ \left[ {{\rm{pVEGFR:pA}}} \right] + {\rm{PTPN}}\ \mathop{\rightleftarrows}\limits_{{{\rm{koff}}_{{\rm{PTPN}}}}}^{{{\rm{kon}}_{{\rm{PTPN}}}}}\ \left[ {{\rm{pVEGFR:pA:PTPN}}} \right]\\ \left[ {{\rm{pVEGFR:pA:PTPN}}} \right]\mathop{\longrightarrow}\limits^{{{\rm{kd}}_{{\rm{PTPN}}}}}{\rm{pVEGFR + [A:PTPN]}}\end{array}$$

for each adapter A and both VEGFRs, where PTPN are phosphatases. Model-predicted adapter phosphorylation in HUVECs shows good agreement to previous experimental data (SI Fig S2). VEGFR1 and VEGFR2 are both modeled for this validation (Fig. 1), as HUVECs express both receptors. Following this validation, we examine adapter–VEGFR1 interactions specifically to determine the VEGFR1 function. See SI Materials and Methods for details.

### Code availability

Model code is available upon request.

### Model assumptions

The following assumptions are used for model development—each assumption is described in SI Materials and Methods

#### Protein concentrations

(1) HUVEC protein concentrations are determined by western blot intensity, relative to a known protein concentration, assuming a linear relationship between protein band intensities (SI Appendix, Table S1). (2) PTPN acts as an “infinite reservoir”; the PTPN concentration is sufficiently high to not be a limiting species in any reaction.

#### Kinetics parameters

(1) Each adapter has the same interaction kinetics (on-rate and off-rate) for both VEGFR1 and VEGFR2 and is the same for all tyrosine sites (SI Table S2). (2) Adapter–VEGFR interaction kinetics are identical to adapter–EGFR interaction kinetics. (3) If adapter–VEGFR or adapter–EGFR interaction rates are unavailable, we assume that the rates between the SH2 domain of the adapter and a phosphorylated tyrosine kinase fragment is identical to the adapter–VEGFR rates. (4) We assume a 1 pL cell volume, to convert rates from M to molecules/cell.

#### Adapter phosphorylation

(1) All adapter phosphorylation rates (kp) are 0.01/s, so adapter phosphorylation is only dependent on VEGFR interaction kinetics. (2) Adapters do not undergo auto-dephosphorylation and are only dephosphorylated by phosphatases. (3) A generalized phosphatase (PTPN) binds and dephosphorylates all adapters, with the same interaction kinetics and dephosphorylation rate.

#### Predicting cell response from adapter phosphorylation

(1) The degradation cell response is identical to c-Cbl phosphorylation; only c-Cbl contributes to a degradation cell response. (2) Proliferation and migration cell responses are determined by a weighed sum of adapter phosphorylation. (3) Weights are calculated by the contribution each adapter provides toward the specific cell response, as determined experimentally (SI Table S3).

#### Tyrosine site specificity

(1) Multiple adapters can bind a single receptor if the combined size of the adapters is smaller than the available space between tyrosine sites (SI Table S4-S5). (2) Adapters bind the receptor in one-dimension (the *y*-direction). (3) Total adapter sizes are determined by measuring the maximal space the adapter crystal structure occupies in the *y*-direction. (4) The center of an adapter binds a VEGFR tyrosine site; thus, the amount of space a receptor occupies between VEGFR tyrosine sites is half the total adapter size. (5) We measure the average distance between VEGFR amino acids and use that distance to determine the space between VEGFR tyrosine sites. For example, the distance between individual amino acids in VEGFR1 was measured as 0.171 Å/amino acid, so the distance between tyrosine sites Tyr^1242^ and Tyr^1333^ is 15.6 Å.

#### Experimental methods

Experiments were performed in RAW 264.7 macrophages due to their high VEGFR1 expression (SI Fig S3), making them an ideal cell line to study VEGFR1 signaling.

### Reagents and cell culture

Murine RAW 264.7 macrophages were cultured in Dulbecco’s Modified Eagle’s Medium supplemented with 10% fetal bovine serum and 1% penicillin–streptomycin. Cells were maintained in a humidified incubator at 37 °C and 5% CO_2_. Murine VEGF-A_164_ was purchased from BioLegend, and all inhibitors (Wortmannin, U73122, and Imatinib Mesylate) were purchased from Selleckchem. Enzyme-linked immunosorbent assay (ELISA) kits were purchased from Assay Biotechnology. The 3-[4,5-dimethylthiazol-2-yl]-2,5 diphenyl tetrazolium bromide (MTT) Cell Proliferation Assay Kit was purchased from Thermo Fisher Scientific.

### Quantifying protein phosphorylation

RAWs were seeded into a 96-well plate, stimulated with VEGF or any inhibitors for specified times, and the phosphorylated and total proteins of interest (PLC_γ_, PI3K, and Abl) were measured using ELISAs. See SI Materials and Methods for details.

### Cell migration assays

RAWs were seeded into a 12-well plate, scratched with a pipette tip, treated with VEGF or any inhibitors, and imaged at 0 and 24 h to characterize migration. See SI Materials and Methods for details.

### Cell proliferation assays

RAWS were seeded into a 96-well plate, stimulated with VEGF or any inhibitors, and cell proliferation was measured after 24 h using a MTT assay. See SI Materials and Methods for details.

### Flow cytometry

RAWs were labeled with Phycoerythrin (PE)-conjugated monoclonal antibodies specific to VEGFR1 or VEGFR2. Fluorescence given off by PE was captured in flow cytometry and converted to VEGFR level per cell (SI Fig S3). See SI Materials and Methods for details.

### Data availability

Supplementary information includes a more detailed description of computational and experimental methods. Further data are available upon request.

## Electronic supplementary material

Supplementary Material File

Supplementary Figure 1

Supplementary Figure 2

Supplementary Figure 3

Supplementary Figure 4

## Electronic supplementary material

**Supplementary information** accompanies the paper on the *npj Systems Biology and Applications* website (10.1038/s41540-017-0037-9).
