# Supplementary material for: VEGFR1 promotes cell migration and proliferation through PLCγ and PI3K pathways
Source: NPJ Syst Biol Appl. 2017 Dec 19;4:1. doi: 10.1038/s41540-017-0037-9 (PMC5736688; doi:10.1038/s41540-017-0037-9)
Supplement: Supplementary file 1 — Supplementary Material File [file 41540_2017_37_MOESM1_ESM.docx]

**Supporting Information**

**VEGFR1 promotes cell migration and proliferation through PLC_γ_ and PI3K pathways**

Jared C. Weddell^1^, Si Chen^1^, and P. I. Imoukhuede^1^

^1^Department of Bioengineering, University of Illinois at Urbana-Champaign, Urbana, IL 61801, USA

Correspondence:

P. I. Imoukhuede

Department of Bioengineering

University of Illinois at Urbana-Champaign

1304 W Springfield Ave.

3233 Digital Computer Laboratory

Urbana, Illinois 61801
Email: pii@illinois.edu

**SI Materials and Methods**

***Computational Methods***

*Obtaining adapter protein initial concentrations.* Adapter initial concentrations in human umbilical vein endothelial cells (HUVECs) were obtained from Western blot data (SI Table S1). We quantified the adapter intensity, and the intensity of a separate protein that had a known concentration, with ImageJ. The background intensities were measured and subtracted from the adapter known protein intensities. We normalized the adapter and known protein intensities to their respective control loading proteins. We calculated the initial adapter concentration as follows:

$\frac{\text{[adapter]}_{0}}{\text{[protein]}_{0}}\text{=}\frac{\text{I}_{\text{adapter-background}}\text{/I}_{\text{control-background}}}{\text{I}_{\text{protein-background}}\text{/I}_{\text{control-background}}}$ (S1)

where $\text{[adapter]}_{0}$ is the initial adapter concentration, $\text{[protein]}_{0}$is the known protein concentration, and $I$ is the intensity. This is a standard technique for initializing and validating computational models^1–4^.

*Obtaining receptor-adapter interaction rates.* Rates for VEGFR-adapter interactions are obtained from isothermal titration calorimetry or surface plasmon resonance experiments (SI Table S2). If VEGFR-adapter specific interaction kinetics are unavailable, we use the kinetics between the adapter SH2 domain and a phosphorylated tyrosine kinase fragment. We assume adapters bind all VEGFR tyrosine sites with the same rate. Forward rates are implemented in $\text{cell/(molecule}\cdot\text{s)}$, using a conversion factor of $1(\text{M}\cdot\text{s)}^{-1}=1.66\cdot10^{-12}\text{cell/(molecule}\cdot\text{s)}$, based on an assumed 1 pL cell volume.

*Modeling adapter phosphorylation and dephosphorylation.* We assume all adapter phosphorylation rates (kp) are 0.01 s^-1^, so adapter phosphorylation is only dependent on VEGFR interaction kinetics. To account for adapter dephosphorylation, we model phosphatase binding to phosphorylated adapters and subsequent adapter dephosphorylation. We assume all adapters are dephosphorylated by a generalized protein tyrosine phosphatase non-receptor type (PTPN) phosphatase^5^. Furthermore, we assume that the PTPN has the same binding kinetics for every adapter, and that the PTPN concentration is sufficiently high to not limit adapter dephosphorylation (SI Table S2).

*Adapter contribution to overall cell response.* The contribution of each adapter to proliferation and migration were obtained from previous experimental studies (SI Table S3). In these experiments, each adapter was inhibited individually, and the percent of endothelial cell proliferation or migration inhibited in response was quantified. We assume for all experiments that the VEGF and drug treatments saturated all cells present. To calculate overall predicted cell response in our simulations, we weight and sum the response contribution of each adapter as follows:

(S2)

where $R_{cell}$ is the overall cell response (proliferation or migration), $w_{R,i}$ is the amount of regulation adapter$i$ gives to that response, and is the phosphorylation of adapter $i$. The adapter weights are determined through by solving the linear problem

$S_{p}w_{R}=e_{R}$ (S3)

Here, $w_{R}$ is a vector containing the weights each adapter contributes to the cell response $R_{cell}$. $e_{R}$ is a vector containing the experimental cell responses, relative to the no inhibition case (SI Appendix, Table S3). $S_{p}$ is a matrix containing the model predicted phosphorylated adapter integrated responses, for each inhibition scheme. Lastly, the weights for proliferation and migration were normalized such that the migration weights summed to one. As these experimental drug treatments look at VEGF signaling in HUVECs as a whole, i.e. signaling contributions from VEGFR1 and VEGFR2, we use both receptors in the drug treatment simulations.

*Goodness of fit tests.* Models were validated against published empirical data^6–10^ by calculating the chi-squared value $\left(\text{χ}^{2}\right)$ (Fig 3, SI Fig S1). $\text{χ}^{2}$ is calculated as follows:

$\text{χ}^{2}=\sum_{i=1}^{N} \frac{\left(y_{i}-f_{i}\right)^{2}}{f_{i}}$ (S4)

where $y_{i}$ is the empirical measurement at index $i$, $f_{i}$ is the simulated value, and $N$is the total number of empirical measurements. Goodness-of-fit was determined by testing the hypothesis that model predicted adapter phosphorylation differs from experimental adapter phosphorylation at the 0.05 significance level. The hypothesis is rejected, which is interpreted as the model accurately predicting experimental adapter phosphorylation, based on the degrees of freedom (df): $\text{χ}^{2}<0.103$ for df = 2 (SI Fig S1F), $\text{χ}^{2}<0.352$ for df = 3 (SI Fig S1E), $\text{χ}^{2}<0.711$ for df = 4 (Fig 3B-C; SI Fig S1A-B, S1D), $\text{χ}^{2}<1.145$ for df = 5 (Fig 3A), and $\text{χ}^{2}<2.167$ for df = 7 (SI Fig S1C)^11^.

*Modeling adapters binding specific VEGFR tyrosine sites.* We assume that multiple adapters can bind a single receptor if the combined size of the adapters is smaller than the available space between tyrosine sites (i.e. the adapters have enough room to bind). To determine what adapter-tyrosine site distributions are possible, we use three pieces of information: (1) the specific tyrosine sites each adapter binds (SI Table S4), (2) the size of each adapter (SI Table S5), and (3) the space between each tyrosine site. These measurements were performed as follows:

*Determining adapter sizes.* Adapter protein sizes were determined by measuring their crystal structures (SI Table S5). To determine the length each adapter blocks on VEGFR1, the intracellular domain of VEGFR1 was assumed to be 1-dimensional (in the y-direction). Adapter protein crystal structures were then oriented such that they bound, via their SH2 domain, to the 1-dimensional VEGFR1. The largest y-direction size of the crystal structure was then measured. We further assume tyrosine sites are bound by the center of adapter proteins, such that half the adapter protein blocks VEGFR1 in the +y-direction and the other half blocks VEGFR1 in the –y-direction. For example, if an adapter protein is 30 Å, it blocks all tyrosine sites within 15 Å of the tyrosine site it is bound to (Fig 1).

*Determining distance between VEGFR1 tyrosine sites.* To determine the distance between VEGFR tyrosine sites, we measured the average distance between amino acids in the VEGFRs tyrosine kinase domain crystal structure (Fig 1A-B). We oriented the tyrosine kinase domain crystal structure to match our 1-dimensional VEGFR assumption; if the crystal structure contained multiple kinase domains, the tyrosine binding sites fall on a vertical line. The tyrosine kinase domain was measured, and that length was divided by the number of amino acids within the crystal structure to give the distance between individual amino acids. The distance between individual amino acids is multiplied by the number of amino acids between VEGFR tyrosine sites to give the distance between VEGFR tyrosine sites. For example, the distance between individual amino acids in VEGFR1 was measured as 0.171 Å/amino acid, so the distance between tyrosine sites Tyr^1242^ and Tyr^1333^ is 15.6 Å.

*Metrics for model analyses.* Cell response was predicted by quantifying two metrics obtained from model simulations: integrated response and amplitude. The integrated response is the area under the phosphorylation versus time curve, while phosphorylation amplitude is simply the peak phosphorylation an adapter reaches^12,13^. These metrics are commonly used to quanitfy total signal propagation and signal propagation speed, respectively^12^. For example, Oyarzún *et. al.* showed that integrated EGFR responses scales linearly with applied ligand stimulus, suggesting that total signal propagation linearly increases with ligand concentration^14^. Likewise, Schilling *et. al.* found that CFU-E cell proliferation is directly correlated with integrated ERK response^13^. Conversely, Kumar *et. al.* found that quantifying these metrics predicts the cell response itself; the Akt phosphorylation amplitude directs apoptosis, whereas Akt integrated response directs proliferation^12^. Therefore, integrated response and phosphorylation amplitude of signaling molecules allow the cell response to be predicted.

*Monte Carlo simulations*. Sensitivity analyses data are represented as the mean ± standard deviation given by 4,000 Monte Carlo simulations. Each Monte Carlo simulation randomly parameterizes the concentrations of the 12 adapters whose concentrations are not being changed for the sensitivity analysis. The random parameterization is pulled from the experimentally derived adapter concentration mean ± standard deviation (SI Table S1).

***Experimental Methods***

*Quantifying protein phosphorylation.* RAW macrophages were seeded into a 96-well plate and grown to ~80% confluence. The cells were then serum starved overnight with DMEM supplemented with 0.5% FBS and 1% PS, and pretreated with any inhibitor overnight: 100 nM Wortmannin (Anti-PI3K, IC_50_ = 3 nM), 10 µM U73122 (Anti-PLC_ɣ_, IC_50_ = 1 µM), or 6 µM Imatinib Mesylate (Anti-Abl, IC_50_ = 0.6 µM). Cells were then stimulated with VEGF-A_164_ (50 ng/mL) for various time periods, and stimulation was stopped by washing the cells with ice cold TBS. Cells were fixed, quenched, blocked, and incubated at 4^o^C overnight with primary antibodies specific for PI3K p85α/γ phosphorylation at Tyr^467/199^, total PI3K p85α/γ, PLC_ɣ_ phosphorylation at Tyr^783^, total PLC_ɣ_, Abl phosphorylation at Tyr^245^, or total Abl. Corresponding HRP-conjugated secondary antibodies were added to the cells, treated with substrate, and absorbance in each well was read at 450 nm to measure protein concentration. Experiments were independently carried out in triplicate. Data is represented as the mean phosphorylated over mean total protein (p/t) ratio ± standard error of the mean (SEM) for each treatment type and treatment time; here SEM is the sum of the phosphorylated and total protein SEMs. The (p/t) ratio given inhibitor treatment specific to the protein of interest was subtracted as background for each treatment time. For example, the PI3K (p/t) ratio given 30 minutes of VEGF stimulation is subtracted by the PI3K (p/t) ratio given 30 minutes of VEGF + Wortmannin stimulation.

*Cell migration assays.* RAWs were seeded into a 12-well plate and grown to ~90% confluence. The cells were then serum starved overnight. The monolayer was then scratched with a 100 µL pipette tip and washed once with PBS to remove floating cells. After the scratch, wells were treated with 750 µL of the serum starved growth factor media containing VEGF-A_164_ (50 ng/mL), 10 µM Wortmannin, 10 µM U73122, 10 µM Imatinib Mesylate, or a combination of VEGF-A_164_ and an inhibitor. Images of the wounded cell monolayer were taken using a microscope at 0 h and 24 h after scratching. All experiments were independently carried out in triplicate. Cell migration was quantified as the number of cells contained within the total gap area relative to the number of cells immediately after the scratch. Cells were counted within the wound margin using ImageJ.

*Cell proliferation assays.* RAWs were seeded into a 96-well plate and grown to ~50% confluence. The cells were then serum starved overnight. Culture medium was removed and cells were stimulated with fresh serum starved media containing VEGF-A_164_ (50 ng/mL), 10 µM Wortmannin, 10 µM U73122, 10 µM Imatinib Mesylate, or a combination of VEGF-A_164_ and an inhibitor for 24 h. MTT was added to each well and incubated at 37^o^C for 4 hours. SDS-HCl solution was added to each well and incubated for another 4 hours at 37^o^C. The solution in each well was mixed with a pipette, and absorbance was read at 570 nm. All experiments were independently carried out in triplicate.

*Cell harvest for qFlow cytometry*. RAWs were harvested when they reach 80- 90% confluency. Cellstripper^TM^ (Millipore, Billerica, MA), a non-enzymatic cell dissociation solution was applied to RAWs and incubated for 4-7 minutes at 37°C/5% CO_2_. Culture flasks were then tapped gently on the side to dislodge cell adherence. Dissociated RAWs were re-suspended in stain buffer (PBS, bovine serum albumin, sodium azide)^16,17^ and centrifuged at 500 ×g for 5 minutes. Supernatant was removed, and RAWs were re-suspended to a final concentration of 4 x 10^6^ cells/mL in stain buffer.

*Cell staining for flow cytometry*. We added 25 µL aliquots of cells (~1 x 10^5^ cells) to 5 ml polystyrene round-bottom tubes (BD Biosciences, New Jersey). Phycoerythrin (PE)-conjugated monoclonal antibodies were added to each tube at the optimal concentrations: 14 µg/mL for VEGFR1 and VEGFR2, determined by titration (SI Fig S3B). We chose the PE fluorophore as the basis of our quantitative fluorescence measurements, because its high extinction coefficient reduces error due to photobleaching, its fluorescence is not quenched by common biomolecules (e.g., antibodies), its fluorescence is independent of pH, and its size minimizes the possibility of multiple fluorophores conjugated to an antibody^18,19^. Samples were incubated in dark for 40 minutes and kept on ice. Samples were then centrifuged at 500 ×g with 4 mL stain buffer for 4 minutes, and supernatant was removed. This washing step was repeated twice. Washed samples were resuspended in 200 – 300 µL stain buffer. For each culture flask, we did not add PE-conjugated antibodies in 1 – 2 samples and those undergo the same procedures as the labeled samples. Those unlabeled samples were used as control to eliminate cell auto-fluorescence and other background noises.

*Quantitative flow cytometry****.*** The precision and accuracy of qFlow cytometry profiling has been rigorously tested^20–23^. Flow cytometry was performed on a LSR Fortessa (BD) Flow cytometer; BD FACSDIVA software was used for data acquisition, and FlowJo (TreeStar) software was used for data analysis. Sytox Blue (Invitrogen), a live/dead cell stain, was added to each sample at a final concentration of 5 μg/mL prior to placement in the flow cytometer. Sytox Blue was excited with a violet laser (407 nm) and its emission was collected using a 450/50 bandpass filter. We plotted histograms of Sytox Blue fluorescence to identify the live cell population. PE was excited with a yellow-green laser (561 nm) and its emission was collected using a 582/15 bandpass filter. Cells exhibiting little to no Sytox Blue fluorescence were gated as live cells. These gated cells were examined in a plot of forward scatter area (FSC-A) versus side scatter area (SSC-A) to gate the single-cell population. We then collected 8,000 - 10,000 live single cells from each tube based on the gating. For each receptor we collected 2 – 4 biological replicates from 3 independent RAW cultures.

*Statistical analysis: ensemble-averaged data****.*** Quantibrite PE beads (BD) were collected and analyzed under the same compensation and voltage settings as cell fluorescence data. Quantibrite PE beads comprise a combination of polystyrene beads conjugated with different density of PE molecules: low (474 PE molecules/bead), medium-low (5,359 PE molecules/bead), medium-high (23,843 PE molecules/bead), and high (62,336 PE molecules/bead). A calibration curve that translated PE geometric mean to the number of bound molecules was determined using linear regression: y = mx+b, where x=log_10_^Number of PE molecules per bead^, y represented log_10_^PE geometric mean per bead^, and m and b represented the slope and intercept of the linear regression, respectively. Receptor levels for VEGFR1 and VEGFR2 were calculated as described previously^24^.

**Table S1. Model protein concentrations.** Adapter, receptor, and phosphatase concentrations in HUVECs were obtained from the references provided. Adapter model concentrations are the mean of the experimental concentrations. Reference protein indicates the known protein concentration used to determine the adapter concentration. In cases where adapters are the reference protein, the model concentration was used as the known concentration. Cell line and conditions used for the experiment are given.

|  | **Protein** | **Model concentration**  **(molecules/cell)** | **Experimental concentration**  **(molecules/cell)** | **Reference Protein** | **Cell Line** | **Cell Conditions** |
| --- | --- | --- | --- | --- | --- | --- |
|  | VEGFR1 | 9.90·10^2^ | 990 ^25^ | - | HUVEC | Growth media (EGM-2) |
|  | VEGFR2 | 1.89·10^3^ | 1890 ^25^ | - |  |  |
|  | PTPN | 8.00·10^4^ | Estimated | - | - | - |
| Both VEGFRs | Abl | 1.50·10^3^ | 2.11·10^3^  ^6^  1.20·10^3 26^  1.18·10^3 27^ | VEGFR2  VEGFR2  Cav1 | HUVEC | Growth media (M199)  24 h serum starvation  Growth media (EGM-2) |
|  | Cav1 | 2.41·10^3^ | 2.02·10^3^ ^28^  2.80·10^3 29^ | VEGFR2  VEGFR2 |  | Growth media  Growth media (EBM) |
|  | c-Cbl | 1.19·10^3^ | 1.19·10^3 30^ | VEGFR2 |  | Growth media |
|  | Crk | 1.11·10^3^ | 1.48·10^3 6^  7.42·10^2 31^ | VEGFR2  FAK |  | Growth media (M199)  2 h serum starvation |
|  | FAK | 1.38·10^3^ | 1.76·10^3 32^  1.53·10^3 33^  8.43·10^2 34^ | VEGFR2  VEGFR2  VEGFR2 |  | 2 h serum starvation  6 h serum starvation  24 h serum starvation |
|  | Fyn | 8.97·10^2^ | 9.96·10^2 35^  7.98·10^2 31^ | VEGFR2  FAK |  | 24 h serum starvation  2 h serum starvation |
|  | GAP | 1.26·10^3^ | 1.32·10^3 36^  1.09·10^3^ ^37^  1.38·10^3^ ^38^ | VEGFR2  VEGFR2  VEGFR2 |  | 24 h serum starvation  Overnight serum starvation  Overnight serum starvation |
|  | Grb2 | 7.10·10^2^ | 9.53·10^2^ ^6^  4.66·10^2 39^ | VEGFR2  ALK1^a^ |  | Growth media (M199)  Growth media (EBM-2) |
|  | Nck | 7.07·10^3^ | 3.23·10^3 40^  1.09·10^4^ ^41^ | PLCɣ  VEGFR2 |  | 6 h serum starvation  18 h serum starvation |
|  | PI3K^a^ | 8.34·10^2^ | 9.33·10^2 42^  6.07·10^2 43^  9.63·10^2 44^ | VEGFR2  Src  VEGFR2 |  | 24 h serum starvation  Growth media (DMEM/F12)  Growth media (M199) |
|  | PLCɣ | 1.10·10^3^ | 1.03·10^3 45^  1.01·10^3 36^  1.25·10^3 8^ | VEGFR2  VEGFR2  GAP |  | 16 h serum starvation  24 h serum starvation  Overnight serum starvation |
|  | Src | 1.62·10^3^ | 1.99·10^3 32^  1.48·10^3 33^  1.38·10^3 46^ | VEGFR2  VEGFR2  VEGFR2 |  | 2 h serum starvation  6 h serum starvation  Overnight serum starvation |
|  | Sck | 1.35·10^3^ | 1.35·10^3 47^ | VEGFR2 |  | 1 h serum starvation |
| VEGFR2 Only | Shb | 2.14·10^3^ | 2.14·10^3 48,49b^ | GAP |  | 20 min serum starvation |
|  | Shc | 1.84·10^3^ | 1.40·10^3^  ^50^  2.28·10^3^  ^51^ | GAP  VEGFR2 |  | Growth media  Serum starvation |
|  | VRAP | 9.01·10^3^ | 9.01·10^2^  ^52^ | PLCɣ |  | 16 h serum starvation |

^a^PI3K is modeled as the p85α domain
^b^ALK1 concentration in HUVECs was quantified by flow cytometry in ^39^.
^c^To calculate the Shb concentration in HUVECs, the Shb/GAP ratio is assumed to be the same in HUVEC and Jurkat cells.

**Table S2. Computational model kinetics.** Adapter-receptor interaction rates were derived from the references provided. Forward and reverse rates for each adapter are from the same reference. Adapter-receptor interaction rates are assumed to be the same for VEGFR1 and VEGFR2.

|  | Receptor | VEGF-receptor forward rate  (cell/molecule·s) | VEGF-receptor reverse rate  (1/s) | Receptor phosphorylation rate |
| --- | --- | --- | --- | --- |
|  | VEGFR1 | 1.81·10^-5^ | 1.00·10^-3^ ^53^ | Immediate^a^ |
|  | VEGFR2 | 6.02·10^-6^ | 1.00·10^-3^ ^53^ | Immediate^a^ |
|  | Protein | Adapter-receptor forward rate  (cell/molecule·s) | Adapter-receptor  reverse rate  (1/s) | Adapter phosphorylation rate (1/s) |
| Both VEGFRs | Abl | 1.06·10^-7^ | 2.27·10^-3 54^ | 0.01^c^ |
|  | Cav1 | 2.47·10^-8^ | 1.76·10^-3 55^ |  |
|  | c-Cbl | 8.30·10^-7^ | 5.00·10^-3 56^ |  |
|  | Crk | 4.65·10^-8^ | 3.10·10^-3 57^ |  |
|  | FAK | 5.50·10^-7^ | 1.00·10^-2 58^ |  |
|  | Fyn | 1.28·10^-7^ | 1.00·10^-2 59^ |  |
|  | GAP | 1.66·10^-6^ | 2.00·10^-1 60^ |  |
|  | Grb2 | 1.66·10^-5^ | 5.50·10^-1 60^ |  |
|  | Nck | 4.98·10^-8^ | 8.10·10^-1 61^ |  |
|  | PI3K | 1.50·10^-6^ | 2.00·10^-2 62^ |  |
|  | PLCɣ | 9.96·10^-5^ | 2.00·10^-2 63^ |  |
|  | Src | 5.48·10^-7^ | 1.20·10^-3 64^ |  |
|  | Sck | 3.32·10^-9^ | 1.00·10^-1 b^ |  |
| VEGFR2 | Shb | 3.32·10^-9^ | 1.00·10^-1 b^ |  |
|  | Shc | 3.32·10^-9^ | 1.00·10^-1 65^ |  |
|  | VRAP | 1.00·10^-7^ | 1.00·10^-2 52^ |  |
| All Adapters | Protein | Adapter-PTPN forward rate (cell/molecule·s) | Adapter-PTPN reverse rate  (1/s) | PTPN dephosphorylation rate |
|  | PTPN | 8.10·10^-6^ | 1.63 ^3^ | 3.39 ^3^ |

^a^VEGFRs are assumed to phosphorylate immediately upon VEGF binding.

^b^Due to limited information, binding rates of Shb and Sck to VEGFRs are assumed to be the same as Shc, as these adapters are part of the same family.

^c^Phosphorylation rate is assumed the same for all adapters.

**Table S3. Adapter contribution to cell proliferation and migration with VEGF treatment.** Percent decrease in EC proliferation and migration when each adapter is inhibited, given by the provided references. Negative percentages indicate an anti-proliferative or anti-migratory role. The cell type, inhibitor concentration, and inhibitor IC_50_ are given. Proliferation and migration weights used to correlate adapter phosphorylation to cell response are given for each adapter. We assume that only c-Cbl phosphorylation contributes to the activation of cell degradation.

| Adapter | % Proliferation Inhibition | Inhibitor | % Migration Inhibition | Inhibitor | Inhibitor IC­_50_ | Model Proliferation Weight | Model Migration Weight |
| --- | --- | --- | --- | --- | --- | --- | --- |
| Abl | 59% ^66^  (HMVEC) | STI571  10 µM | 10% ^66^ (HMVEC) | STI571  10 µM | 0.8 µM ^67^ | 1.8·10^-1^ | -8.0·10^-2^ |
| Cav1 | -42% ^68^  (HUVEC) | Cav1 transfection | 46% ^69^  (HUVEC) | siRNA | - | -6.5·10^-2^ | 3.3·10^-1^ |
| c-Cbl | Negligible^a^ | - | Negligible^a^ | - | - | 0 | 0 |
| Crk | Negligible^a^ | - | 60% ^70^  (HUVEC) | Mutation | - | 0 | 3.1·10^-1^ |
| FAK | < 1% ^71^  (HUVEC) | siRNA | 30% ^72^  (HUVEC) | siRNA | - | 0 | 1.5·10^-2^ |
| Fyn | 15% ^73^  (HRMEC) | siRNA | -25% ^73^  (HRMEC) | siRNA | - | 1.4·10^-2^ | -2.1·10^-1^ |
| GAP | 63% ^74^ (HUVEC) | Fasudil  10 µM | 50% ^74^  (HUVEC) | Fasudil  10 µM | 1.2 µM ^75^ | 2.1·10^-2^ | 1.2·10^-2^ |
| Grb2 | 40% ^76^  (HUVEC) | C90  0.3 µM | 47% ^76^  (HUVEC) | C90  0.3 µM | 150 nM ^77^ | 1.3·10^-1^ | 1.1·10^-1^ |
| Nck | Negligible^a^ | - | 40% ^78^  (HUVEC) | shRNA | - | 0 | 5.1·10^-2^ |
| PI3K | 28% ^79^  (HUVEC) | LY294002  10 µM | 48% ^80^  (HUVEC) | LY294002  10 µM | 0.5 µM ^81^ | 1.1·10^-1^ | 1.7·10^-1^ |
| PLC_ɣ_ | 50% ^82^  (HUVEC) | U73122  10 µM | 65% ^80^  (HUVEC) | U73122  6 µM | 0.8 µM ^83^ | 1.9·10^-1^ | 2.5·10^-1^ |
| Sck | Negligible^a^ | - | Negligible^a^ | - | - | 0 | 0 |
| Shb | Negligible^a^ | - | Negligible^a^ | - | - | 0 | 0 |
| Shc | Negligible ^84^ | shRNA | Negligible ^84^ | shRNA | - | 0 | 0 |
| Src | 29% ^85^  (HUVEC) | M475271  3 µM | 68% ^85^  (HUVEC) | M475271  3 µM | 25 nM ^86^ | 5.8·10^-2^ | 1.7·10^-1^ |
| VRAP | -9% ^10^  (HUVEC) | siRNA | 44% ^10^  (HUVEC) | siRNA | - | -1.4·10^-1^ | 1.6·10^-1^ |

HUVEC – Human umbilical vein endothelial cell; HMVEC – Human microvascular endothelial cell;

HRMEC – Human retinal microvascular endothelial cell;
^a^Not identified, we assume the adapter contribution is negligible to other adapters

**Table S4. Adapter-VEGFR tyrosine site interaction references.** References indicate were the adapter-VEGFR tyrosine site interaction was derived. These interactions are either empirically observed, or provide information leading to an assumed interaction. For example, showing an interaction between an adapter and an amino acid chain that correlates to a VEGFR tyrosine site. Several adapter-VEGFR interactions were also empirically observed without specifying the specific tyrosine site; assumptions for these tyrosine sites are given in the footnotes, with all references relating to the assumption in the table.

| **VEGFR1**  **Interaction** | **Reference** | **VEGFR1**  **Interaction** | **Reference** | **VEGFR2**  **Interaction** | **Reference** | **VEGFR2**  **Interaction** | **Reference** |
| --- | --- | --- | --- | --- | --- | --- | --- |
| Y794-PLC_ɣ_ | 87 | Y1213-Cav1 | 88,89g | Y801-PLC_ɣ_ | 87 | Y1175-Abl | 90 |
| Y794-PI3K | 91 | Y1213-FAK | 89,92h | Y801-PI3K | 93 | Y1175-Shc | 62,94,95i |
| Y1169-PLC_ɣ_ | 96 | Y1242-PLC_ɣ_ | 87 | Y951-PLC_ɣ_ | 91,97 | Y1175-Shb | 95 |
| Y1169-GAP | 98,99a | Y1333-PLC_ɣ_ | 100 | Y951-VRAP | 10 | Y1175-Sck | 101 |
| Y1169-Src | 98,99b | Y1333-Nck | 100,102 | Y1008-PLC_ɣ_ | 103 | Y1214-PI3K | 102,104j |
| Y1169-Abl | 98,99,105c | Y1333-Crk | 100 | Y1059-PLC_ɣ_ | 106,107 | Y1214-Fyn | 89 |
| Y1169-Sck | 101,108d | Y1333-c-Cbl | 109,110 | Y1059-Src | 111 | Y1214-Nck | 89 |
| Y1213-PLC_ɣ_ | 100 | - | - | Y1059-GAP | 111 | Y1214-FAK | 112 |
| Y1213-PI3K | 113 | - | - | Y1059-c-Cbl | 111,114 | Y1214-Crk | 70,89k |
| Y1213-Fyn | 89 | - | - | Y1175-PLC_ɣ_ | 90,115 | Y1214-Src | 89,111 |
| Y1213-Nck | 102 | - | - | Y1175-Src | 111 | Y1214-GAP | 116 |
| Y1213-Src | 89,117e | - | - | Y1175-PI3K | 93 | Y1214-Cav1 | 116,118 |
| Y1213-GAP | 89,119f | - | - | Y1175-Grb2 | 120 | Y1305-FAK | 112,121 |
| Y1213-Grb2 | 100 | - | - | Y1175-GAP | 122,123 | - | - |

^a^ Assumed based on GAP interaction at VEGFR2-Y1175, and VEGFR1-Y1169 homology with VEGFR2-Y1175.

^b^ Assumed based on Src interaction at VEGFR2-Y1175, and VEGFR1-Y1169 homology with VEGFR2-Y1175.

^c^ Assumed based on Abl interaction at VEGFR2-Y1175, and VEGFR1-Y1169 homology with VEGFR2-Y1175.

^d^ Assumed based on Sck interaction at VEGFR2-Y1175, and VEGFR1-Y1169 homology with VEGFR2-Y1175.

^e^ Assumed based on Src interaction at VEGFR2-Y1214, and VEGFR1-Y1213 homology with VEGFR2-Y1214.

^f^ Assumed based on GAP interaction at VEGFR2-Y1214, and VEGFR1-Y1213 homology with VEGFR2-Y1214.

^g^ Assumed based on Cav1 interaction at VEGFR2-Y1214, and VEGFR1-Y1213 homology with VEGFR2-Y1214.

^h^ Assumed based on FAK interaction at VEGFR2-Y1214, and VEGFR1-Y1213 homology with VEGFR2-Y1214.

^i^ Assumed based on Shb interaction at VEGFR2-Y1175, and Shc homology with Shb.

^j^ Assumed based on PI3K interaction at VEGFR1-Y1213, and VEGFR2-Y1214 homology with VEGFR1-Y1213.

^k^ Assumed based on Nck interaction at VEGFR2-Y1214, and Crk homology with Nck.

**Table S5. Adapter sizes.** Sizes of each adapter protein used in the VEGFR1-adapter protein models. Note that entire crystal structures are rarely available, and so the domain each crystal structure contains is given. The segment of amino acids (AA) contained in the crystal structure is given compared to the total AA in the protein sequence. The Protein Data Bank and reference for each crystal structure are given if available.

| **Adapter^a^** | **Domain** | **Crystal AA** | **Total AA** | **Size (Å)** | **PDB Entry** | **Reference** |
| --- | --- | --- | --- | --- | --- | --- |
| Abl | SH2 | 38-512 | 1130 | 31.34 | 3T04 | 124 |
| c-Cbl | SH2, N-term | 1-323 | 474 | 53.57 | 3VRR | 125 |
| Crk | SH2 | 1-204 | 304 | 31.39 | 2EYY | 126 |
| FAK | FA | 891-1052 | 1052 | 33.55 | 4NY0 | 127 |
| Fyn | SH2 | 143-248 | 537 | 33.97 | 1AOT | 128 |
| GAP | SH2 | 341-446 | 1047 | 39.13 | 2GSB | - |
| Grb2 | SH2 | 53-163 | 217 | 31.31 | 4P9V | 129 |
| Nck | SH2 | 281-377 | 377 | 30.41 | 2CI9 | 61 |
| PI3K | SH2 | 617-724 | 724 | 32.57 | 1H9O | 130 |
| PLC_ɣ_ | SH2 | 545-790 | 1290 | 34.29 | 4FBN | 131 |
| Src | SH2 | 144-252 | 536 | 32.76 | 4F5B | 132 |
| Shc^b^ | SH2 | 147-311 | 583 | 37.28 | 1SHC | 133 |

^a^No crystal structure for VRAP or Cav1 available. We assume they have a 30 Å lower size limit.
^b^No crystal structures for Shb and Sck available. We assume they are the same size as Shc as they are part of the same family.

**Table S6. RAW macrophage adapter concentrations.** Adapter, receptor, and phosphatase concentrations in RAW 264.7 macrophages were derived from the references provided. reference protein indicates the known protein concentration that was used to determine the adapter concentration. Concentrations for all reference proteins are provided. The cell line and conditions used for the experimental measurements are also given. For cell condition, starvation time and growth media is given, if available. Adapter concentrations are given as mean ± standard error of the mean, from the experimental measurement.

| **Protein** | **Model concentration**  **(molecules/cell)** | **Reference Protein** | **Cell Line** | **Cell**  **Condition** |
| --- | --- | --- | --- | --- |
| VEGFR1 | 4.82·10^3^ ± 1.12·10^2^ | Measured | RAW | Growth media (DMEM) |
| VEGFR2 | 1.77·10^3^ ± 1.32·10^2^ | Measured |  |  |
| PTPN | 8.00·10^4^ | Estimated | - | - |
| Abl | 3.20·10^3^ ± 5.92·10^2^ ^134^ | FAK | RAW | Growth media (DMEM) |
| Cav1 | 4.29·10^3^ ± 7.94·10^2^ ^135^ | p38 |  | Growth media (DMEM) |
| c-Cbl | 2.48·10^3^ ± 4.58·10^2^ ^136^ | p38 |  | Growth media (DMEM) |
| Crk | 2.71·10^3^ ± 5.00·10^2^ ^134^ | FAK |  | Growth media (DMEM) |
| FAK | 3.30·10^3^ ± 6.10·10^2^ ^92^ | VEGFR1 |  | Growth media (alpha-MEM) |
| Fyn^a^ | 2.70·10^3^ ± 5.00·10^2^ ^137^ | FAK |  | Growth media (RPMI 1640) |
| GAP | 9.90·10^3^ ± 1.84·10^3^ ^138^ | ERK |  | Growth media |
| Grb2 | 7.13·10^3^ ± 1.32·10^3^ ^138^ | ERK |  | Growth media |
| Nck | 3.80·10^3^ ± 7.02·10^2^ ^139^ | VASP |  | Growth media (alpha-MEM) |
| PI3K | 2.28·10^3^ ± 4.22·10^2^ ^140^ | Akt |  | Growth media (DMEM) |
| PLCɣ | 2.28·10^3^ ± 4.22·10^2^ ^141^ | IKBα |  | Growth media (RPMI 1640) |
| Sck^b^ | 0 | - |  | - |
| Shb^b^ | 0 | - |  | - |
| Shc | 1.08·10^4^ ± 2.00·10^3^ ^138^ | ERK |  | Growth media |
| Src | 2.61·10^3^ ± 4.82·10^2^  ^142^ | FAK |  | Growth media (DMEM) |
| VRAP^b^ | 2.00·10^3^ | - |  | - |
| Akt | 2.28·10^3^ ± 4.22·10^2 143^ | p38 |  | Growth media (DMEM) |
| ERK | 5.21·10^3^ ± 9.68·10^2 143^ | p38 |  | Growth media (DMEM) |
| IKBα | 2.19·10^3^ ± 4.04·10^2 144^ | p38 |  | Growth media (DMEM) |
| p38 | 2.24·10^3^ ± 4.15·10^2 134^ | FAK |  | Growth media (DMEM) |
| VASP | 2.38·10^3^ ± 4.40·10^2 145^ | IKBα |  | Growth media (DMEM) |

^a^The Fyn concentration was unavailable. Instead, we assume the Fyn concentration is equal to the Lyn concentration, as they are a part of the same family.
^b^The adapter concentration is unavailable. Since VRAP and Shb do not bind to VEGFR1, and we found Sck to not significantly direct VEGFR1 signaling, their concentrations are not essential for determining VEGFR1 signaling. Thus, we assume these concentrations are 0 molecules/cell, within the range of the other adapter concentrations.

**Fig S1. PLC and PI3K are preferentially activated at Tyr^794^ on VEGFR1.** The (A) integrated responses (area under the activation-time curves) and (B) phosphorylation amplitudes were quantified for all adapters stemming from each VEGFR1 tyrosine site.

**Fig S2. Both nonspecific and specific VEGFR1 site models predict relative adapter phosphorylation.** Fitting model predicted adapter phosphorylation versus time to relative adapter phosphorylation through both VEGFR1 and VEGFR2. Experimental data was normalized so the maximum adapter phosphorylation is 1. Adapter phosphorylation was simulated for the same time length given by experimental measurements, and the maximum predicted adapter phosphorylation was normalized to 1. Model accuracy is tested with the Χ^2^ goodness-of-fit test^11^. References for experimental data are: (A) Crk^6^, (B) Nck^6^, (C) FAK^7^, (D) PLC_ɣ_^8^, (E) Src^9^, and (F) VRAP^10^.

**Fig S3. VEGFR1 and VEGFR2 quantification on RAWs.** (A) Membrane VEGFR1 and VEGFR2 levels on RAWs were measured by quantitative flow cytometry. Data is represented as mean ± standard error of the mean. (B) Saturation curves of VEGFR1 and VEGFR2 antibodies on RAWs show that all receptors are labeled, ensuring accurate quantification.

**Fig S4. Contribution of VEGFR1 and VEGFR2 to RAW macrophage signaling.** The contribution of VEGFR1 and VEGFR2 to adapter phosphorylation and RAW migration and proliferation are given. For each adapter and cell response, the phosphorylation amplitude (pA_amp_) and integrated response (pA_int_) contributed by VEGR1 and VEGFR2 specifically are given. Note that Shb, Shc, and VRAP are VEGFR2-specific adapters (Fig 1), and the concentration of Sck and Shb are assumed zero in RAWs (SI Table S6).

**SI Appendix References**

1. Tan, C. W., Gardiner, B. S., Hirokawa, Y., Smith, D. W. & Burgess, A. W. Analysis of Wnt signaling β-catenin spatial dynamics in HEK293T cells. *BMC Syst. Biol.* **8,** 44 (2014).

2. Nicklas, D. & Saiz, L. Computational modelling of Smad-mediated negative feedback and crosstalk in the TGF-β superfamily network. *J. R. Soc. Interface* **10,** 20130363 (2013).

3. Tan, W. H., Popel, A. S. & Mac Gabhann, F. Computational model of VEGFR2 pathway to ERK activation and modulation through receptor trafficking. *Cell Signal* **25,** 2496–2510 (2013).

4. Wu, Y. *et al.* Modeling the mitotic regulatory network identifies highly efficient anti-cancer drug combinations. *Mol. BioSyst.* **11,** 497–505 (2015).

5. Wu, C. *et al.* Systematic identification of SH3 domain-mediated human protein-protein interactions by peptide array target screening. *Proteomics* **7,** 1775–85 (2007).

6. Anselmi, F. *et al.* c-ABL modulates MAP kinases activation downstream of VEGFR-2 signaling by direct phosphorylation of the adaptor proteins GRB2 and NCK1. *Angiogenesis* **15,** 187–197 (2012).

7. Qi, J. H. & Claesson-Welsh, L. VEGF-Induced Activation of Phosphoinositide 3-Kinase Is Dependent on Focal Adhesion Kinase. *Exp. Cell Res.* **263,** 173–182 (2001).

8. Bhattacharya, R. *et al.* Distinct role of PLCbeta3 in VEGF-mediated directional migration and vascular sprouting. *J. Cell Sci.* **122,** 1025–34 (2009).

9. Ha, C. H., Bennett, A. M. & Jin, Z. G. A novel role of vascular endothelial cadherin in modulating c-Src activation and downstream signaling of vascular endothelial growth factor. *J. Biol. Chem.* **283,** 7261–70 (2008).

10. Matsumoto, T. *et al.* VEGF receptor-2 Y951 signaling and a role for the adapter molecule TSAd in tumor angiogenesis. *EMBO J.* **24,** 2342–2353 (2005).

11. Hogg, R. V & Tanis, E. A. *Probability and statistical inference*. (2015).

12. Kumar, D., Srikanth, R., Ahlfors, H., Lahesmaa, R. & Rao, K. V. S. Capturing cell-fate decisions from the molecular signatures of a receptor-dependent signaling response. *Mol. Syst. Biol.* **3,** 150 (2007).

13. Schilling, M. *et al.* Theoretical and experimental analysis links isoform-specific ERK signalling to cell fate decisions. *Mol. Syst. Biol.* **5,** 334 (2009).

14. Oyarzún, D. A. *et al.* The EGFR demonstrates linear signal transmission. *Integr. Biol.* **6,** 736–42 (2014).

15. Birtwistle, M. R. *et al.* Ligand-dependent responses of the ErbB signaling network: experimental and modeling analyses. *Mol. Syst. Biol.* **3,** 144 (2007).

16. Imoukhuede, P. I. & Popel, A. S. A. Quantification and cell-to-cell variation of vascular endothelial growth factor receptors. *Exp. Cell Res.* **317,** 955–965 (2011).

17. Roxworthy, B. J. *et al.* Plasmonic optical trapping in biologically relevant media. *PLoS One* **9,** e93929 (2014).

18. He, B. *et al.* Grand challenges in interfacing engineering with life sciences and medicine. *IEEE Trans. Biomed. Eng.* **60,** 589–98 (2013).

19. Burrell, R. a, McGranahan, N., Bartek, J. & Swanton, C. The causes and consequences of genetic heterogeneity in cancer evolution. *Nature* **501,** 338–45 (2013).

20. Willett, C. G. *et al.* *Direct evidence that the VEGF-specific antibody bevacizumab has antivascular effects in human rectal cancer.* *Nat. Med.* **10,** (2004).

21. Bergers, G., Song, S., Meyer-Morse, N., Bergsland, E. & Hanahan, D. Benefits of targeting both pericytes and endothelial cells in the tumor vasculature with kinase inhibitors. *J. Clin. Invest.* **111,** 1287–1295 (2003).

22. Erber, R. *et al.* Combined inhibition of VEGF and PDGF signaling enforces tumor vessel regression by interfering with pericyte-mediated endothelial cell survival mechanisms. *FASEB J.* **18,** 338–340 (2004).

23. Casanovas, O., Hicklin, D. J., Bergers, G. & Hanahan, D. Drug resistance by evasion of antiangiogenic targeting of VEGF signaling in late-stage pancreatic islet tumors. *Cancer Cell* **8,** 299–309 (2005).

24. Chen, S. *et al.* qFlow cytometry-based receptoromic screening: a high-throughput quantification approach informing biomarker selection and nanosensor development. *Methods Mol Biol* **(in press),** (2016).

25. Chen, S., Guo, X., Imarenezor, O. & Imoukhuede, P. Quantification of VEGFRs, NRP1, and PDGFRs on Endothelial Cells and Fibroblasts Reveals Serum, Intra-Family Ligand, and Cross-Family Ligand Regulation. *Cell. Mol. Bioeng.* **8,** 383–403 (2015).

26. Chislock, E. M., Ring, C. & Pendergast, A. M. Abl kinases are required for vascular function, Tie2 expression, and angiopoietin-1–mediated survival. *Proc. Natl. Acad. Sci. U. S. A.* **110,** 12432–12437 (2013).

27. Takeuchi, K. *et al.* AMP-dependent Kinase Inhibits Oxidative Stress-induced Caveolin-1 Phosphorylation and Endocytosis by Suppressing the Dissociation between c-Abl and Prdx1 Proteins in Endothelial Cells. *J. Biol. Chem.* **288,** 20581–20591 (2013).

28. Ikeda, S. *et al.* Novel Role of ARF6 in Vascular Endothelial Growth Factor–Induced Signaling and Angiogenesis. *Circ. Res.* **96 ,** 467–475 (2005).

29. Fang, L. *et al.* Control of angiogenesis by AIBP-mediated cholesterol efflux. *Nature* **498,** 118–122 (2013).

30. Song, H. *et al.* Novel epsin-VEGFR2 interactions facilitated by c-Cbl ubiquitination of epsin and VEGFR2 regulate VEGFR2 signaling and physiological and pathological angiogenesis. in *Vasculata* Poster Presentation (2014).

31. Ohmori, T. *et al.* Gi-mediated Cas Tyrosine Phosphorylation in Vascular Endothelial Cells Stimulated with Sphingosine 1-Phosphate: POSSIBLE INVOLVEMENT IN CELL MOTILITY ENHANCEMENT IN COOPERATION WITH Rho-MEDIATED PATHWAYS . *J. Biol. Chem.* **276 ,** 5274–5280 (2001).

32. Jean, C. *et al.* Inhibition of endothelial FAK activity prevents tumor metastasis by enhancing barrier function. *J. Cell Biol.* **204,** 247–63 (2014).

33. Chen, X. L. *et al.* VEGF-induced vascular permeability is mediated by FAK. *Dev. Cell* **22,** 146–157 (2012).

34. Herzog, B., Pellet-Many, C., Britton, G., Hartzoulakis, B. & Zachary, I. C. VEGF binding to NRP1 is essential for VEGF stimulation of endothelial cell migration, complex formation between NRP1 and VEGFR2, and signaling via FAK Tyr407 phosphorylation. *Mol. Biol. Cell* **22,** 2766–2776 (2011).

35. Sinha, S. *et al.* Dopamine regulates phosphorylation of VEGF receptor 2 by engaging Src-homology-2-domain-containing protein tyrosine phosphatase 2. *J. Cell Sci.* **122,** 3385–92 (2009).

36. Hoeppner, L. H. *et al.* RhoC maintains vascular homeostasis by regulating VEGF-induced signaling in endothelial cells. *J. Cell Sci.* **128,** 3556–3568 (2015).

37. Lai, L. *et al.* Plumbagin inhibits tumour angiogenesis and tumour growth through the Ras signalling pathway following activation of the VEGF receptor-2. *Br. J. Pharmacol.* **165,** 1084–1096 (2012).

38. Yoshioka, K. *et al.* Endothelial PI3K-C2[alpha], a class II PI3K, has an essential role in angiogenesis and vascular barrier function. *Nat Med* **18,** 1560–1569 (2012).

39. Tabata, T. *et al.* Induction of an Epithelial Integrin &#x3b1;v&#x3b2;6 in Human Cytomegalovirus-Infected Endothelial Cells Leads to Activation of Transforming Growth Factor-&#x3b2;1 and Increased Collagen Production. *Am. J. Pathol.* **172,** 1127–1140 (2016).

40. Arroyo, J., Torry, R. J. & Torry, D. S. Deferential regulation of placenta growth factor (PlGF)-mediated signal transduction in human primary term trophoblast and endothelial cells. *Placenta* **25,** 379–86 (2004).

41. Dubrac, A. *et al.* Targeting NCK-Mediated Endothelial Cell Front-Rear Polarity Inhibits NeovascularizationCLINICAL PERSPECTIVE. *Circulation* **133,** 409–421 (2016).

42. Li, S. *et al.* VEGFR tyrosine kinase inhibitor II (VRI) induced vascular insufficiency in zebrafish as a model for studying vascular toxicity and vascular preservation. *Toxicol. Appl. Pharmacol.* **280,** 408–20 (2014).

43. Yue, G. G.-L. *et al.* Novel PI3K/AKT targeting anti-angiogenic activities of 4-vinylphenol, a new therapeutic potential of a well-known styrene metabolite. *Sci. Rep.* **5,** 11149 (2015).

44. Coon, B. G. *et al.* Intramembrane binding of VE-cadherin to VEGFR2 and VEGFR3 assembles the endothelial mechanosensory complex. *J. Cell Biol.* **208 ,** 975–986 (2015).

45. Xiong, Y. *et al.* Hypertensive stretch regulates endothelial exocytosis of Weibel-Palade bodies through VEGF receptor 2 signaling pathways. *Cell Res.* **23,** 820–34 (2013).

46. Han, S.-W. *et al.* DICAM inhibits angiogenesis via suppression of AKT and p38 MAP kinase signalling. *Cardiovasc. Res.* **98,** 73–82 (2013).

47. Ratcliffe, K. E. *et al.* Sck is expressed in endothelial cells and participates in vascular endothelial growth factor-induced signaling. *Oncogene* **21,** 6307–6316 (2002).

48. Lindholm, C. K., Henriksson, M. L., Hallberg, B. & Welsh, M. Shb links SLP-76 and Vav with the CD3 complex in Jurkat T cells. *Eur. J. Biochem.* **269,** 3279–3288 (2002).

49. Aviva_Systems_Biology. *SHB Antibody*. (2014).

50. Ma, Z., Liu, Z., Wu, R.-F. & Terada, L. S. p66Shc restrains Ras hyperactivation and suppresses metastatic behavior. *Oncogene* **29,** 5559–5567 (2010).

51. Oshikawa, J. *et al.* Novel role of p66Shc in ROS-dependent VEGF signaling and angiogenesis in endothelial cells. *Am. J. Physiol. - Hear. Circ. Physiol.* **302,** H724–H732 (2012).

52. Wu, L. W. *et al.* VRAP is an adaptor protein that binds KDR, a receptor for vascular endothelial cell growth factor. *J. Biol. Chem.* **275,** 6059–6062 (2000).

53. Weddell, J. C. & Imoukhuede, P. I. Quantitative characterization of cellular membrane-receptor heterogeneity through statistical and computational modeling. *PLoS One* **9,** e97271 (2014).

54. Xiong, X. *et al.* Allosteric inhibition of the nonMyristoylated c-Abl tyrosine kinase by phosphopeptides derived from Abi1/Hssh3bp1. *Biochim. Biophys. Acta* **1783,** 737–47 (2008).

55. Huang, J. H. *et al.* Identification of the HIV-1 gp41 core-binding motif in the scaffolding domain of caveolin-1. *J. Biol. Chem.* **282,** 6143–52 (2007).

56. Huang, L. *et al.* Simulating EGFR-ERK signaling control by scaffold proteins KSR and MP1 reveals differential ligand-sensitivity co-regulated by Cbl-CIN85 and endophilin. *PLoS One* **6,** e22933 (2011).

57. Matsuda, M. *et al.* Interaction between the amino-terminal SH3 domain of CRK and its natural target proteins. *J. Biol. Chem.* **271,** 14468–14472 (1996).

58. Arold, S. T. *et al.* The role of the Src homology 3-Src homology 2 interface in the regulation of Src kinases. *J. Biol. Chem.* **276,** 17199–205 (2001).

59. Solheim, S. A. *et al.* Interactions between the Fyn SH3-domain and adaptor protein Cbp/PAG derived ligands, effects on kinase activity and affinity. *FEBS J.* **275,** 4863–74 (2008).

60. Schoeberl, B., Eichler-Jonsson, C., Gilles, E. D. & Muller, G. Computational modeling of the dynamics of the MAP kinase cascade activated by surface and internalized EGF receptors. *Nat. Biotech* **20,** 370–375 (2002).

61. Frese, S. *et al.* The phosphotyrosine peptide binding specificity of Nck1 and Nck2 Src homology 2 domains. *J. Biol. Chem.* **281,** 18236–18245 (2006).

62. Tan, W. H., Popel, A. S. & Mac Gabhann, F. Computational Model of Gab1/2-Dependent VEGFR2 Pathway to Akt Activation. *PLoS One* **8,** e67438 (2013).

63. Kholodenko, B. N., Demin, O. V., Moehren, G. & Hoek, J. B. Quantification of short term signaling by the epidermal growth factor receptor. *J. Biol. Chem.* **274,** 30169–30181 (1999).

64. Payne, G., Shoelson, S. E., Gish, G. D., Pawson, T. & Walsh, C. T. Kinetics of p56lck and p60src Src homology 2 domain binding to tyrosine-phosphorylated peptides determined by a competition assay or surface plasmon resonance. *Proc. Natl. Acad. Sci. U. S. A.* **90,** 4902–6 (1993).

65. Zhou, M.-M. *et al.* Binding Affinities of Tyrosine-phosphorylated Peptides to the COOH-terminal SH2 and NH-terminal Phosphotyrosine Binding Domains of Shc . *J. Biol. Chem.* **270 ,** 31119–31123 (1996).

66. Yan, W., Bentley, B. & Shao, R. Distinct angiogenic mediators are required for basic fibroblast growth factor- and vascular endothelial growth factor-induced angiogenesis: the role of cytoplasmic tyrosine kinase c-Abl in tumor angiogenesis. *Mol. Biol. Cell* **19,** 2278–2288 (2008).

67. Roumiantsev, S. *et al.* Clinical resistance to the kinase inhibitor STI-571 in chronic myeloid leukemia by mutation of Tyr-253 in the Abl kinase domain P-loop. *Proc. Natl. Acad. Sci. U. S. A.* **99,** 10700–10705 (2002).

68. Fang, K. *et al.* Overexpression of caveolin-1 inhibits endothelial cell proliferation by arresting the cell cycle at G0/G1 phase. *Cell Cycle* **6,** 199–204 (2007).

69. Tahir, S. A., Park, S. & Thompson, T. C. Caveolin-1 regulates VEGF-stimulated angiogenic activities in prostate cancer and endothelial cells. *Cancer Biol. Ther.* **8,** 2284–2294 (2014).

70. Stoletov, K. V, Gong, C. & Terman, B. I. Nck and Crk mediate distinct VEGF-induced signaling pathways that serve overlapping functions in focal adhesion turnover and integrin activation. *Exp. Cell Res.* **295,** 258–268 (2004).

71. Bryant, P. W., Zheng, Q. & Pumiglia, K. M. Focal adhesion kinase is a phospho-regulated repressor of Rac and proliferation in human endothelial cells. *Biol. Open* **1,** 723–730 (2012).

72. Le Boeuf, F., Houle, F., Sussman, M. & Huot, J. Phosphorylation of focal adhesion kinase (FAK) on Ser732 is induced by rho-dependent kinase and is essential for proline-rich tyrosine kinase-2–mediated phosphorylation of FAK on Tyr407 in response to vascular endothelial growth factor. *Mol. Biol. Cell* **17,** 3508–3520 (2006).

73. Werdich, X. Q. & Penn, J. S. Src, Fyn and Yes play differential roles in VEGF-mediated endothelial cell events. *Angiogenesis* **8,** 315–26 (2005).

74. Yin, L. *et al.* Fasudil inhibits vascular endothelial growth factor-induced angiogenesis in vitro and in vivo. *Mol. Cancer Ther.* **6,** 1517–1525 (2007).

75. Rikitake, Y. *et al.* Inhibition of Rho kinase (ROCK) leads to increased cerebral blood flow and stroke protection. *Stroke* **36,** 2251–2257 (2005).

76. Soriano, J. V *et al.* Inhibition of angiogenesis by growth factor receptor bound protein 2-Src homology 2 domain binding antagonists. *Mol. Cancer Ther.* **3,** 1289–1300 (2004).

77. Giubellino, A. *et al.* Inhibition of tumor metastasis by a growth factor receptor bound protein 2 Src homology 2 domain-binding antagonist. *Cancer Res.* **67,** 6012–6016 (2007).

78. Chaki, S. P. *et al.* Nck enables directional cell migration through the coordination of polarized membrane protrusion with adhesion dynamics. *J. Cell Sci.* **126,** 1637–49 (2013).

79. Kim, H., Ko, H., Choi, S. & Seo, D. Anti-angiogenic effects of Siegesbeckia glabrescens are mediated by suppression of the Akt and p70S6K-dependent signaling pathways. *Oncol. Rep.* **33,** 699–704 (2015).

80. Short, S. M. *et al.* Inhibition of endothelial cell migration by thrombospondin-1 type-1 repeats is mediated by β1 integrins. *J. Cell Biol.* **168,** 643–653 (2005).

81. Arcaro, A. & Wymann, M. P. Wortmannin is a potent phosphatidylinositol 3-kinase inhibitor: the role of phosphatidylinositol 3,4,5-trisphosphate in neutrophil responses. *Biochem. J.* **296 (Pt 2),** 297–301 (1993).

82. Zeng, H., Dvorak, H. F. & Mukhopadhyay, D. Vascular permeability factor (VPF)/vascular endothelial growth factor (VEGF) receptor-1 down-modulates VPF/VEGF receptor-2-mediated endothelial cell proliferation, but not migration, through phosphatidylinositol 3-kinase-dependent pathways. *J. Biol. Chem.* **276,** 26969–26979 (2001).

83. Tatrai, A., Lee, S. K. & Stern, P. H. U-73122, a phospholipase C antagonist, inhibits effects of endothelin-1 and parathyroid hormone on signal transduction in UMR-106 osteoblastic cells. *Biochim Biophys Acta* **1224,** 575–82 (1994).

84. Sweet, D. T., Chen, Z., Wiley, D. M., Bautch, V. L. & Tzima, E. The adaptor protein Shc integrates growth factor and ECM signaling during postnatal angiogenesis. *Blood* **119,** 1946–1955 (2012).

85. Ali, N. *et al.* The novel Src kinase inhibitor M475271 inhibits VEGF-induced vascular endothelial-cadherin and beta-catenin phosphorylation but increases their association. *J. Pharmacol. Sci.* **102,** 112–120 (2006).

86. Rivat, C. *et al.* Synergistic cooperation between the AP-1 and LEF-1 transcription factors in activation of the matrilysin promoter by the src oncogene: implications in cellular invasion. *FASEB J* **17,** 1721–3 (2003).

87. Cunningham, S. A., Arrate, M. P., Brock, T. A. & Waxham, M. N. Interactions of FLT-1 and KDR with phospholipase C gamma: identification of the phosphotyrosine binding sites. *Biochem. Biophys. Res. Commun.* **240,** 635–9 (1997).

88. Podar, K. *et al.* Caveolin-1 is required for vascular endothelial growth factor-triggered multiple myeloma cell migration and is targeted by bortezomib. *Cancer Res* **64,** 7500–7506 (2004).

89. Lamalice, L., Houle, F. & Huot, J. Phosphorylation of Tyr1214 within VEGFR-2 triggers the recruitment of Nck and activation of Fyn leading to SAPK2/p38 activation and endothelial cell migration in response to VEGF. *J. Biol. Chem.* **281,** 34009–20 (2006).

90. Chislock, E. M. & Pendergast, A. M. Abl Family Kinases Regulate Endothelial Barrier Function <italic>In Vitro</italic> and in Mice. *PLoS One* **8,** e85231 (2013).

91. Ahmad, S. *et al.* Direct evidence for endothelial vascular endothelial growth factor receptor-1 function in nitric oxide-mediated angiogenesis. *Circ. Res.* **99,** 715–22 (2006).

92. Matsumoto, Y. *et al.* Possible Involvement of the Vascular Endothelial Growth Factor-Flt-1-Focal Adhesion Kinase Pathway in Chemotaxis and the Cell Proliferation of Osteoclast Precursor Cells in Arthritic Joints. *J. Immunol.* **168,** 5824–5831 (2002).

93. Dayanir, V. *et al.* Identification of Tyrosine Residues in Vascular Endothelial Growth Factor Receptor-2/FLK-1 Involved in Activation of Phosphatidylinositol 3-Kinase and Cell Proliferation. *J Biol Chem* **276,** 17686–17692 (2001).

94. Zanetti, A. *et al.* Vascular endothelial growth factor induces Shc association with vascular endothelial cadherin: A potential feedback mechanism to control vascular endothelial growth factor receptor-2 signaling. *Arterioscler. Thromb. Vasc. Biol.* **22,** 617–622 (2002).

95. Xu, Q. *et al.* Human CD4+CD25low adaptive T regulatory cells suppress delayed-type hypersensitivity during transplant tolerance. *J. Immunol.* **178,** 3983–3995 (2007).

96. Sawano, A., Takahashi, T., Yamaguchi, S. & Shibuya, M. The phosphorylated 1169-tyrosine containing region of flt-1 kinase (VEGFR-1) is a major binding site for PLCgamma. *Biochem. Biophys. Res. Commun.* **238,** 487–91 (1997).

97. Wu, L. W. *et al.* Utilization of distinct signaling pathways by receptors for vascular endothelial cell growth factor and other mitogens in the induction of endothelial cell proliferation. *J. Biol. Chem.* **275,** 5096–5103 (2000).

98. Shibuya, M. Vascular Endothelial Growth Factor Receptor Family Genes: When Did the Three Genes Phylogenetically Segregate? *Biol. Chem.* **383,** 1573–1579 (2002).

99. Vieira, J. M., Ruhrberg, C. & Schwarz, Q. VEGF receptor signaling in vertebrate development. *Organogenesis* **6,** 97–106 (2010).

100. Ito, N., Wernstedt, C., Engstrom, U. & Claesson-Welsh, L. Identification of vascular endothelial growth factor receptor-1 tyrosine phosphorylation sites and binding of SH2 domain-containing molecules. *J. Biol. Chem.* **273,** 23410–23418 (1998).

101. Warner, A. J., Lopez-Dee, J., Knight, E. L., Feramisco, J. R. & Prigent, S. A. The Shc-related adaptor protein, Sck, forms a complex with the vascular-endothelial-growth-factor receptor KDR in transfected cells. *Biochem. J.* **347,** 501–509 (2000).

102. Igarashi, K. *et al.* Tyrosine 1213 of Flt-1 Is a Major Binding Site of Nck and SHP-2. *Biochem. Biophys. Res. Commun.* **246,** 95–99 (1998).

103. Meyer, R. D., Latz, C. & Rahimi, N. Recruitment and Activation of Phospholipase Cγ1 by Vascular Endothelial Growth Factor Receptor-2 Are Required for Tubulogenesis and Differentiation of Endothelial Cells. *J. Biol. Chem.* **278,** 16347–16355 (2003).

104. Caron, C. *et al.* Non-redundant roles of the Gab1 and Gab2 scaffolding adapters in VEGF-mediated signalling, migration, and survival of endothelial cells. *Cell. Signal.* **21,** 943–953 (2009).

105. Maru, Y., Hirosawa, H. & Shibuya, M. An oncogenic form of the Flt-1 kinase has a tubulogenic potential in a sinusoidal endothelial cell line. *Eur. J. Cell Biol.* **79,** 130–43 (2000).

106. Napione, L. *et al.* Unraveling the influence of endothelial cell density on VEGF-A signaling. *Blood* **119,** 5599–607 (2012).

107. Zeng, H., Sanyal, S. & Mukhopadhyay, D. Tyrosine Residues 951 and 1059 of Vascular Endothelial Growth Factor Receptor-2 (KDR) Are Essential for Vascular Permeability Factor/Vascular Endothelial Growth Factor-induced Endothelium Migration and Proliferation, Respectively. *J. Biol. Chem.* **276,** 32714–32719 (2001).

108. Igarashi, K., Shigeta, K., Isohara, T., Yamano, T. & Uno, I. Sck interacts with KDR and Flt-1 via its SH2 domain. *Biochem. Biophys. Res. Commun.* **251,** 77–82 (1998).

109. Kobayashi, S. *et al.* The c-Cbl/CD2AP complex regulates VEGF-induced endocytosis and degradation of Flt-1 (VEGFR-1). *FASEB J.* **18,** 929–31 (2004).

110. Raikwar, N. S., Liu, K. Z. & Thomas, C. P. N-Terminal Cleavage and Release of the Ectodomain of Flt1 Is Mediated via ADAM10 and ADAM 17 and Regulated by VEGFR2 and the Flt1 Intracellular Domain. *PLoS One* **9,** e112794 (2014).

111. Meyer, R. D., Sacks, D. B. & Rahimi, N. IQGAP1-Dependent Signaling Pathway Regulates Endothelial Cell Proliferation and Angiogenesis. *PLoS One* **3,** e3848 (2008).

112. Qi, J. H. & Claesson-Welsh, L. VEGF-induced activation of phosphoinositide 3-kinase is dependent on focal adhesion kinase. *Exp. Cell Res.* **263,** 173–182 (2001).

113. Yu, Y. *et al.* Direct identification of a major authophosphorylation site on vascular endothelial growth factor receptor Flt-1 that mediates phosphatidylinositol 3’-kinase binding. *Biochem J* **358,** 465–472 (2001).

114. Singh, A. J. *et al.* A critical role for the E3-ligase activity of c-Cbl in VEGFR-2-mediated PLCγ1 activation and angiogenesis. *Proc. Natl. Acad. Sci.* **104,** 5413–5418 (2007).

115. Takahashi, T., Yamaguchi, S., Chida, K. & Shibuya, M. A single autophosphorylation site on KDR/Flk-1 is essential for VEGF-A-dependent activation of PLC-[gamma] and DNA synthesis in vascular endothelial cells. *EMBO J* **20,** 2768–2778 (2001).

116. Lamalice, L., Houle, F. F., Jourdan, G. & Huot, J. Phosphorylation of tyrosine 1214 on VEGFR2 is required for VEGF-induced activation of Cdc42 upstream of SAPK2//p38. *Oncogene* **23,** 434–445 (2004).

117. Chou, M. T., Wang, J. & Fujita, D. J. Src kinase becomes preferentially associated with the VEGFR, KDR/Flk-1, following VEGF stimulation of vascular endothelial cells. *BMC Biochem.* **3,** 32 (2002).

118. Tiwari, A., Jung, J.-J., Inamdar, S. M., Nihalani, D. & Choudhury, A. The myosin motor Myo1c is required for VEGFR2 delivery to the cell surface and for angiogenic signaling. *Am. J. Physiol. Heart Circ. Physiol.* **304,** H687–96 (2013).

119. Jones, M. C. *et al.* VEGFR1 (Flt1) Regulates Rab4 Recycling to Control Fibronectin Polymerization and Endothelial Vessel Branching. *Traffic* **10,** 754–766 (2009).

120. Koch, S. & Claesson-Welsh, L. Signal Transduction by Vascular Endothelial Growth Factor Receptors. *Cold Spring Harb. Perspect. Med.* **2,** a006502 (2012).

121. Bahary, N. *et al.* Duplicate VegfA genes and orthologues of the KDR receptor tyrosine kinase family mediate vascular development in the zebrafish. *Blood* **110,** 3627–3636 (2007).

122. Park, S. Y., Shi, X., Pang, J., Yan, C. & Berk, B. C. Thioredoxin-interacting protein mediates sustained VEGFR2 signaling in endothelial cells required for angiogenesis. *Arterioscler. Thromb. Vasc. Biol.* **33,** 737–743 (2013).

123. Jopling, H. M. *et al.* Rab GTPase Regulation of VEGFR2 Trafficking and Signaling in Endothelial Cells. *Arter. Thromb Vasc Biol* **29,** 1119–1124 (2009).

124. Grebien, F. *et al.* Targeting the SH2-kinase interface in Bcr-Abl inhibits leukemogenesis. *Cell* **147,** 306–319 (2011).

125. Takeshita, K. *et al.* Structural flexibility regulates phosphopeptide-binding activity of the tyrosine kinase binding domain of Cbl-c. *J. Biochem.* **152,** 487–495 (2012).

126. Kobashigawa, Y. *et al.* Structural basis for the transforming activity of human cancer-related signaling adaptor protein CRK. *Nat Struct Mol Biol* **14,** 503–510 (2007).

127. Brami-Cherrier, K. *et al.* FAK dimerization controls its kinase-dependent functions at focal adhesions. *EMBO J.* **33,** 356–370 (2014).

128. Mulhern, T. D., Shaw, G. L., Morton, C. J., Day, A. J. & Campbell, I. D. The SH2 domain from the tyrosine kinase Fyn in complex with a phosphotyrosyl peptide reveals insights into domain stability and binding specificity. *Structure* **5,** 1313–23 (1997).

129. Myslinski, J. M., Clements, J. H. & Martin, S. F. Protein–ligand interactions: Probing the energetics of a putative cation–π interaction. *Bioorg. Med. Chem. Lett.* **24,** 3164–3167 (2014).

130. Pauptit, R. A. *et al.* NMR trial models: experiences with the colicin immunity protein Im7 and the p85alpha C-terminal SH2-peptide complex. *Acta Crystallogr D Biol Crystallogr* **57,** 1397–404 (2001).

131. Bunney, T. D. *et al.* Structural and functional integration of the PLCγ interaction domains critical for regulatory mechanisms and signaling deregulation. *Struct. England1993)* **20,** 2062–2075 (2012).

132. Kaneko, T. *et al.* Superbinder SH2 domains act as antagonists of cell signaling. *Sci. Signal.* **5,** ra68–ra68 (2012).

133. Zhou, M. M. *et al.* Structure and ligand recognition of the phosphotyrosine binding domain of Shc. *Nature* **378,** 584–592 (1995).

134. Greuber, E. K. & Pendergast, A. M. Abl Family Kinases Regulate FcgR-Mediated Phagocytosis in Murine Macrophages. *J Immunol* **189,** 5382–92 (2012).

135. Wu, C.-C. *et al.* OxLDL upregulates caveolin-1 expression in macrophages: Role for caveolin-1 in the adhesion of oxLDL-treated macrophages to endothelium. *J. Cell. Biochem.* **107,** 460–472 (2009).

136. Kundu, M. *et al.* A TNF- and c-Cbl-dependent FLIPS-degradation pathway and its function in Mycobacterium tuberculosis-induced macrophage apoptosis. *Nat Immunol* **10,** 918–926 (2009).

137. Hsieh, M.-Y. *et al.* The Inducible Nitric-oxide Synthase (iNOS)/Src Axis Mediates Toll-like Receptor 3 Tyrosine 759 Phosphorylation and Enhances Its Signal Transduction, Leading to Interferon-β Synthesis in Macrophages. *J. Biol. Chem.* **289,** 9208–9220 (2014).

138. Wolfson, M., Yang, C.-P. H. & Horwitz, S. B. Taxol induces tyrosine phosphorylation of SHC and its association with GRB2 in murine raw 264.7 cells. *Int. J. Cancer* **70,** 248–252 (1997).

139. Coppolino, M. G. *et al.* Evidence for a molecular complex consisting of Fyb/SLAP, SLP-76, Nck, VASP and WASP that links the actin cytoskeleton to Fcγ receptor signalling during phagocytosis. *J. Cell Sci.* **114,** 4307–4318 (2001).

140. Zhang, Y. *et al.* Schisandrin B inhibits cell growth and induces cellular apoptosis and autophagy in mouse hepatocytes and macrophages: implications for its hepatotoxicity. *Drug Des Devel Ther* **9,** 2001–2027 (2015).

141. Chiang, C.-Y., Veckman, V., Limmer, K. & David, M. Phospholipase Cγ-2 and Intracellular Calcium Are Required for Lipopolysaccharide-induced Toll-like Receptor 4 (TLR4) Endocytosis and Interferon Regulatory Factor 3 (IRF3) Activation. *J. Biol. Chem.* **287,** 3704–3709 (2012).

142. Heckel, T. *et al.* Src-dependent repression of ARF6 is required to maintain podosome-rich sealing zones in bone-digesting osteoclasts. *Proc. Natl. Acad. Sci. U. S. A.* **106,** 1451–1456 (2009).

143. McGuire, V. A. *et al.* Cross Talk between the Akt and p38α Pathways in Macrophages Downstream of Toll-Like Receptor Signaling. *Mol. Cell. Biol.* **33 ,** 4152–4165 (2013).

144. Liu, Y., Su, W., Wang, S. & Li, P. Naringin inhibits chemokine production in an LPS‑induced RAW 264.7 macrophage cell line. *Mol Med Rep* **6,** 1343–50 (2012).

145. Tateya, S. *et al.* Endothelial NO/cGMP/VASP Signaling Attenuates Kupffer Cell Activation and Hepatic Insulin Resistance Induced by High-Fat Feeding. *Diabetes* **60,** 2792–2801 (2011).
