# Supplementary figures and images for: VEGFR1 promotes cell migration and proliferation through PLCγ and PI3K pathways
Source: NPJ Syst Biol Appl. 2017 Dec 19;4:1. doi: 10.1038/s41540-017-0037-9 (PMC5736688; doi:10.1038/s41540-017-0037-9)

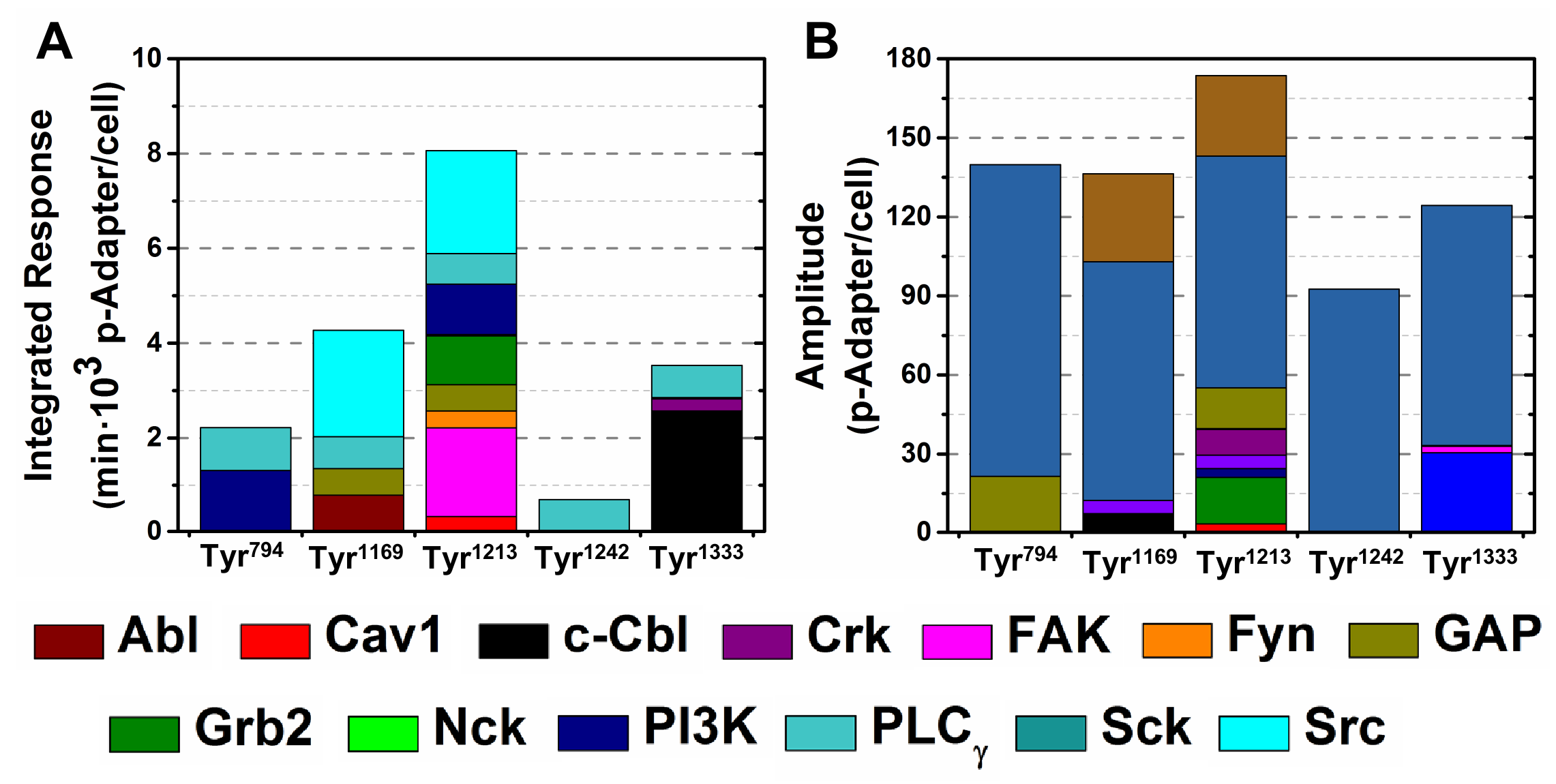

Supplement: Supplementary file 2 — Supplementary Figure 1 [file 41540_2017_37_MOESM2_ESM.tif]

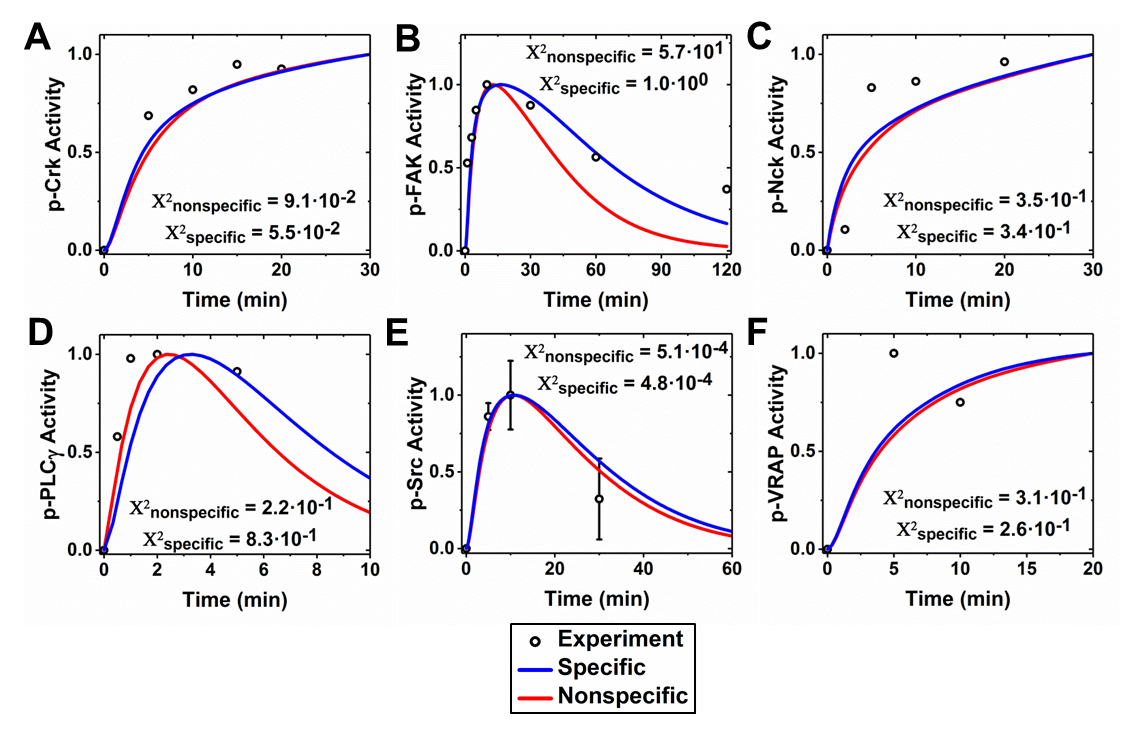

Supplement: Supplementary file 3 — Supplementary Figure 2 [file 41540_2017_37_MOESM3_ESM.tif]

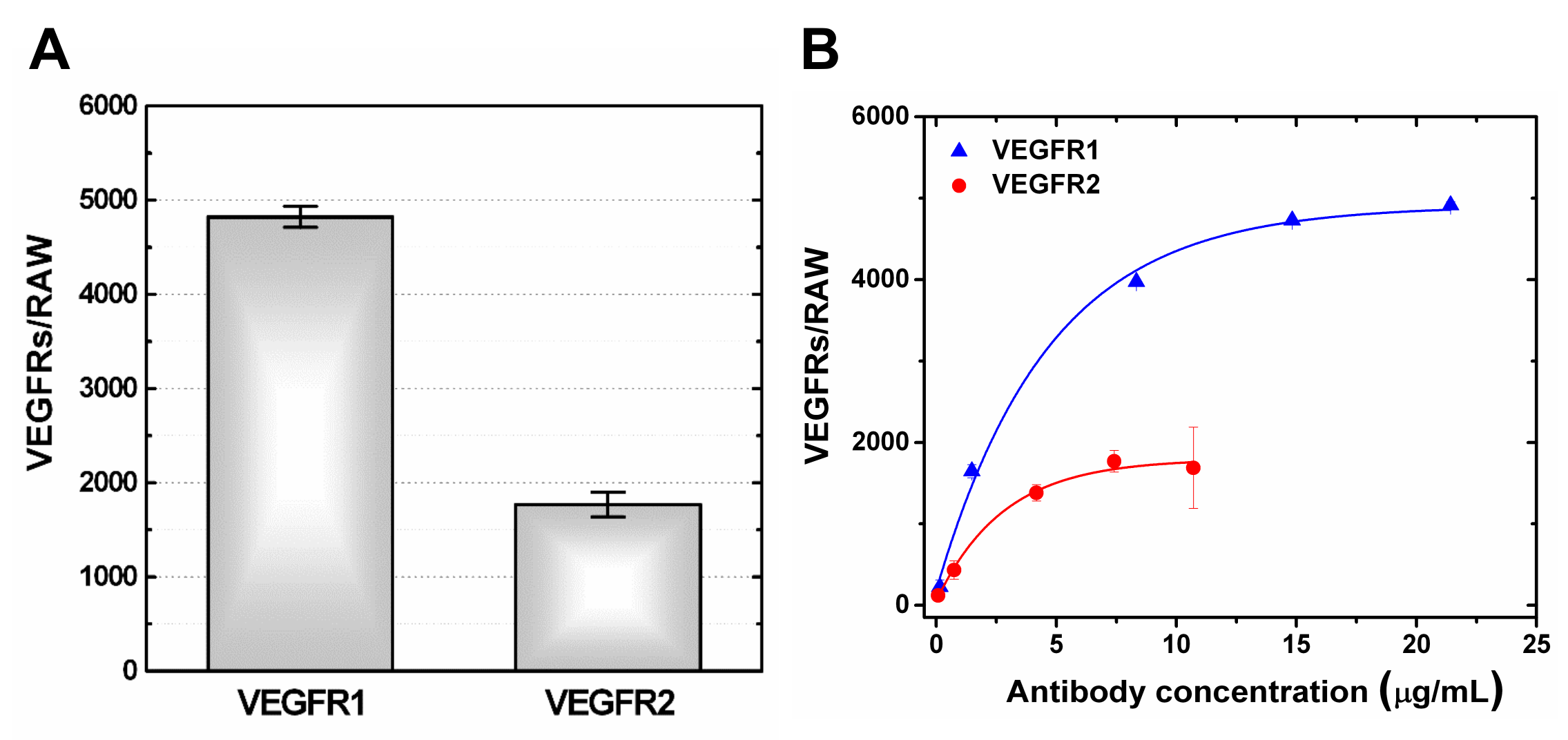

Supplement: Supplementary file 4 — Supplementary Figure 3 [file 41540_2017_37_MOESM4_ESM.tif]

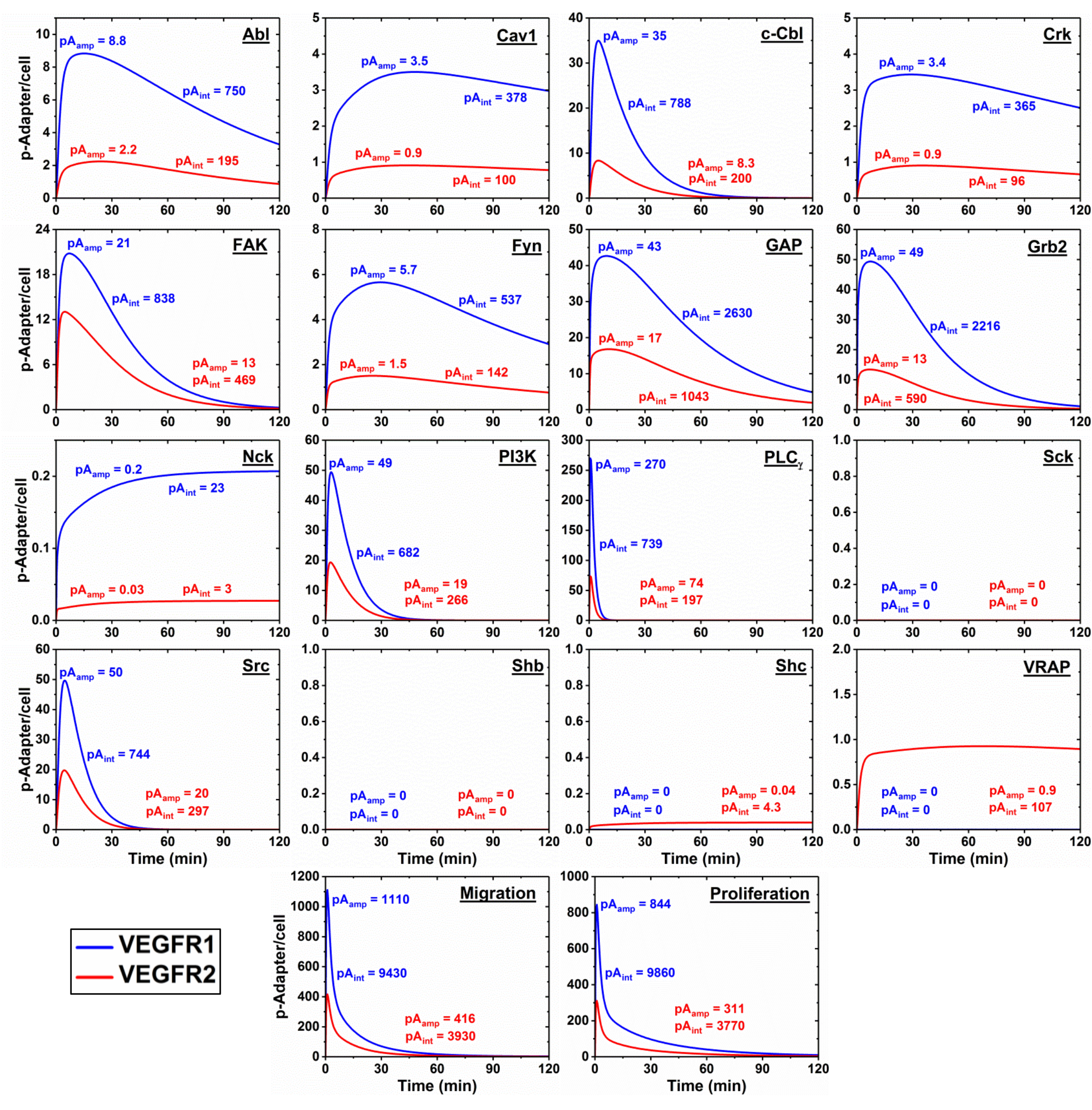

Supplement: Supplementary file 5 — Supplementary Figure 4 [file 41540_2017_37_MOESM5_ESM.tif]
